# Reconciling spatial and temporal soil moisture effects on afternoon rainfall

**DOI:** 10.1038/ncomms7443

**Published:** 2015-03-05

**Authors:** Benoit P. Guillod, Boris Orlowsky, Diego G. Miralles, Adriaan J. Teuling, Sonia I. Seneviratne

**Affiliations:** 1 Institute for Atmospheric and Climate Science, Department of Environmental Systems Science, ETH Zurich, 8092 Zurich, Switzerland; 2 Department of Earth Sciences, VU University Amsterdam, Amsterdam 1081 HV, The Netherlands; 3 Laboratory of Hydrology and Water Management, Department of Forest and Water Management, Ghent University, B-9000 Ghent, Belgium; 4 Hydrology and Quantitative Water Management Group, Department of Environmental Sciences, Wageningen University, Wageningen 6708PA, The Netherlands

## Abstract

Soil moisture impacts on precipitation have been strongly debated. Recent observational evidence of afternoon rain falling preferentially over land parcels that are drier than the surrounding areas (negative spatial effect), contrasts with previous reports of a predominant positive temporal effect. However, whether spatial effects relating to soil moisture heterogeneity translate into similar temporal effects remains unknown. Here we show that afternoon precipitation events tend to occur during wet and heterogeneous soil moisture conditions, while being located over comparatively drier patches. Using remote-sensing data and a common analysis framework, spatial and temporal correlations with opposite signs are shown to coexist within the same region and data set. Positive temporal coupling might enhance precipitation persistence, while negative spatial coupling tends to regionally homogenize land surface conditions. Although the apparent positive temporal coupling does not necessarily imply a causal relationship, these results reconcile the notions of moisture recycling with local, spatially negative feedbacks.

## Abstract (web summary)

The sign of soil moisture–precipitation feedback has been strongly debated. Here, the authors show that rain tends to fall where soils are drier than their surroundings, but on days with overall wet and heterogeneous conditions, explaining the apparent contradictions between recent studies.

## Graphical abstract

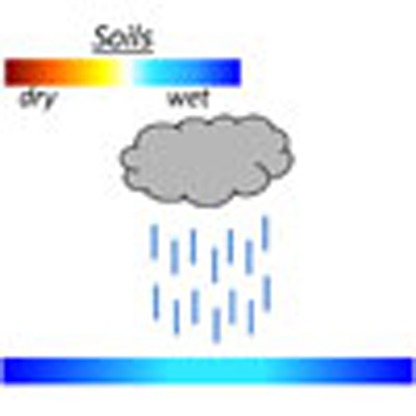

Land climate interactions play an important role in the climate system^1^, in particular in transitional climate regions, where soil moisture influences the partitioning of the energy available at the land surface into sensible and latent heat fluxes^2^. Surface turbulent fluxes may influence precipitation directly via moisture input to the atmosphere (moisture recycling^3^), as well as indirectly, via boundary-layer dynamics^4^ and mesoscale circulations^5^. Moisture recycling is expected to lead to a positive feedback, that is, more precipitation induced by wet conditions. The indirect effect via mesoscale circulations, on the other hand, may lead to a negative effect^5^. Finally, the indirect effect via boundary-layer dynamics can theoretically lead to feedbacks of both signs depending on atmospheric conditions^4^,^6^,^7^. Studies in the 1990s and 2000s have mostly identified positive coupling mechanisms using models or reanalyses^8^,^9^,^10^. However, ref. 11 has recently suggested a strong dominance of negative coupling mechanisms in observations contrasting with a strong positive coupling in Global Climate Models. This negative coupling could be consistent with negative indirect effects via soil-moisture-induced mesoscale circulations^5^ or boundary-layer dynamics. The apparent contradiction between these latter results and previous studies has led to a recent debate on the dominant sign of soil moisture–precipitation feedbacks.

Since the sign of the feedback exhibited by climate models has been shown to be sensitive to the parameterization of convection^12^,^13^, the use of models with explicit convection or—if possible—the direct inference of the underlying relationships from observations, is essential to avoid parameterization-dependent results. Recently, global data sets of soil moisture, evaporation and precipitation from satellite remote sensing have become available and provide a unique opportunity to study the soil moisture–precipitation coupling mechanisms globally. However, observational analyses are impaired by the difficulty of establishing a causal relationship^1^,^14^,^15^. The spatial analysis from ref. 11 attempts to overcome this issue by comparing, for a given day, soil moisture at locations with and without rain, to mitigate the impacts of atmospheric persistence on the relationship. Spatial analyses, however, are specifically designed to investigate local, indirect effects and one cannot exclude that they might reflect processes that differ from the traditional understanding of soil moisture–precipitation feedbacks. Indeed, spatial gradients of soil moisture might be largely independent of large-scale soil moisture availability. Coupling mechanisms via mesoscale circulations might thus interact with moisture recycling and other effects at larger scales^5^. However, the different methodologies and data sets employed in previous studies hamper direct comparison of the results.

The first comparison of spatial and temporal effects of soil moisture on precipitation is presented here, using long-term global remote-sensing-based data sets of precipitation and morning soil moisture, available over the period 2002–2011 at 3-hourly and daily time steps, respectively, and at a spatial resolution of 0.25° (see Methods). To directly compare spatial and temporal approaches, we use the method by ref. 11 to identify afternoon precipitation events, and we compare temporal and spatial structures of pre-event morning soil moisture to non-event days. In particular, we examine whether rain is more likely on days when soils are wetter or drier than climatological conditions, at locations with drier or wetter soils than the surrounding areas, and on days with large or small spatial soil moisture variability. Using this consistent analysis framework, we find that globally, afternoon rain is more likely at locations that are dry compared with the surrounding area (that is, negative spatial correlation), on days that are wet compared with the mean seasonal cycle (that is, positive temporal correlation), and on days with more heterogeneous soil moisture conditions than expected for a region and season. These results demonstrate the coexistence of positive temporal and negative spatial relationships within the same region and based on the same data. Although a positive temporal correlation does not necessarily imply a causal relationship, our results potentially reconcile the notions of positive temporal soil moisture–precipitation coupling with local, spatially negative feedbacks. We further propose physical mechanisms by which these apparently contradictory processes could coexist.

## Results

### Analysis of precipitation events

In this study, a precipitation event domain is defined as 5 × 5 grid cells (0.25° × 0.25° each, that is, 1.25° × 1.25° in total) centred at a location of local afternoon precipitation maxima (Lmax) with at least 4 mm of accumulated rain. The event domain is denoted Levt, while Lmin is the location of precipitation minimum within Levt. Events that occur in days with morning precipitation are excluded from the analysis, as our focus is on the triggering of new rainfall events. Similarly, events located over fixed features that may influence precipitation location such as complex topography or water bodies are excluded, and only months when convective conditions dominate are retained for the analysis (see Methods). For each event, morning temporal soil moisture anomalies *S*′ relative to the mean seasonal cycle are analysed at various locations or combinations of these. More precisely, we define three metrics based on three combinations (*Y*^s^, *Y*^t^ and *Y*^h^) of *S*′ at locations Lmax, Lmin and/or Levt: a spatial metric, defined as 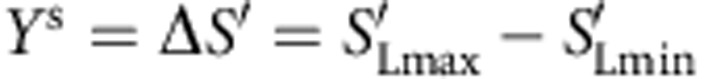, which compares soil moisture at location of precipitation maximum versus precipitation minimum; a temporal metric, defined as 
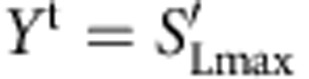
, which quantifies anomalies of soil moisture at the event locations relative to the seasonal cycle (or, alternatively, using either 
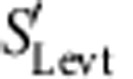
 or 
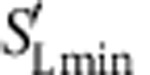
 instead of 
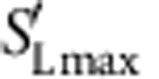
, see Supplementary Discussion); and a heterogeneity metric, defined as 
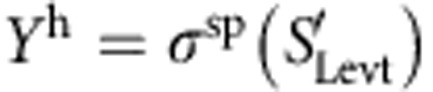
 (the spatial s.d. of the 25 *S*′ values within Levt), which quantifies spatial soil moisture heterogeneity.

The method to quantify the strength of a relationship between a variable *Y* and precipitation events follows ref. 11 (therein, *Y*=*Y*^s^), and is applied for fixed 5° × 5° boxes. For each event, we compute *Y* and denote it as *Y*_e_. Then we define a control sample based on data for non-event days, *Y*_c_ (using the same locations on non-event days). All values of *Y*_e_ and *Y*_c_ within a 5° × 5° box are pooled together, and we compute the difference in *Y* values between the event and control sample, *δ*_e_(*Y*)=mean(*Y*_e_)−mean(*Y*_c_). We then compare *δ*_e_(*Y*) to typical values of *δ*(*Y*) obtained from bootstrapping (see Methods Summary) by displaying the quantile of typical values corresponding to *δ*_e_(*Y*). In other words, we compare *Y* from event days with non-event days and measure the strength of this difference with expectations from resampling.

### Spatial relationships

**Figure 1:**
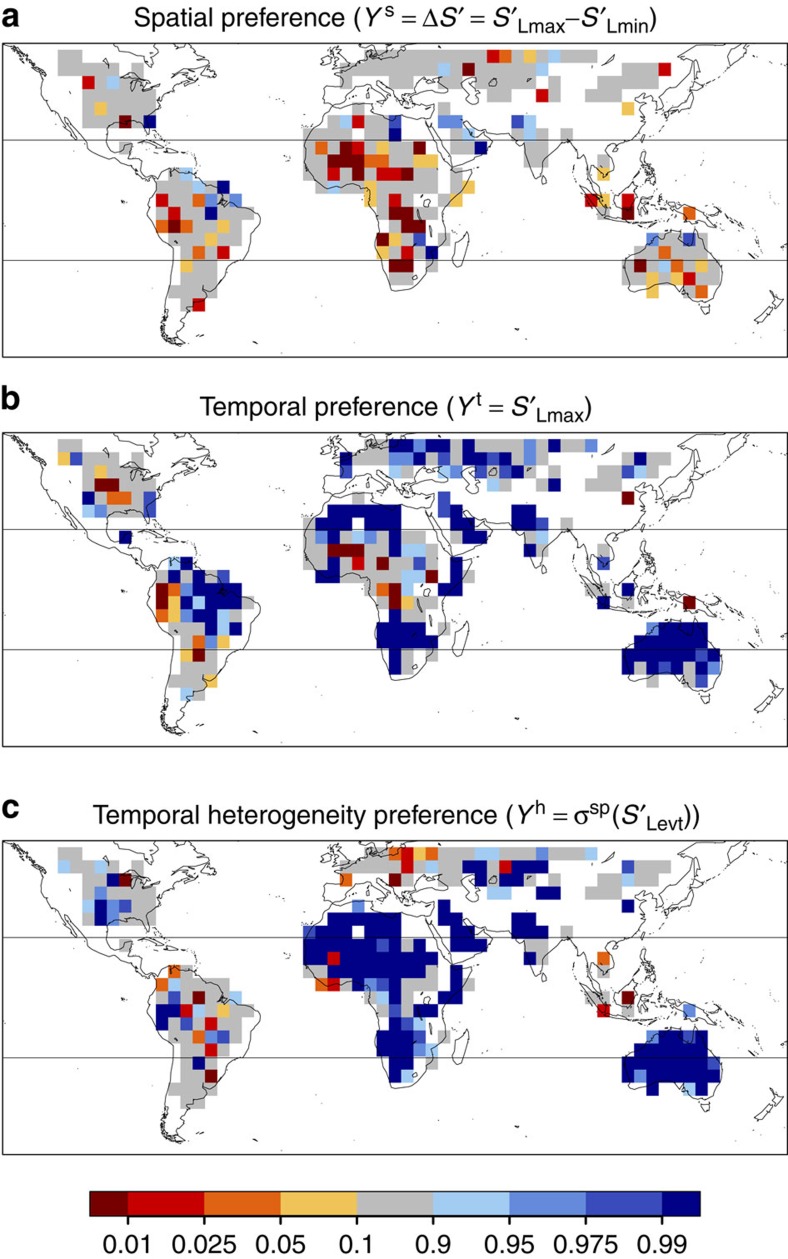
Preferences for afternoon precipitation over soil moisture anomalies. (**a**) Spatial, (**b**) temporal and (**c**) heterogeneity preference. Quantile of the coupling metric *δ*_e_(*Y*)=mean(*Y*_e_)−mean(*Y*_c_) under the Null hypothesis that no coupling exists, where *Y* is (**a**) 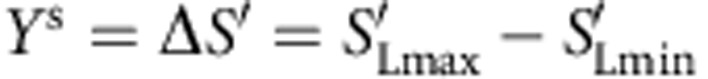, the difference in *S*′ between the location of rainfall maximum and the location of rainfall minimum, (**b**) 
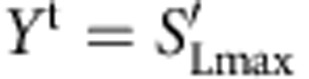
 and (**c**) 
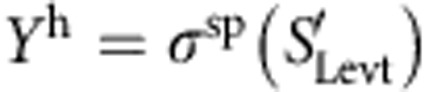
. Low (high) quantiles indicate where *Y* is lower (higher) than expected. Horizontal black lines indicate the latitudes at which different months are included in the analysis (see Methods). Grey shading indicates non-significant relationships, grid cells with <25 events are left white. Results from various data sets are shown in Supplementary Figs 1–4, 7 and 8.

The results of the spatial analysis are displayed in Fig. 1a (see also Supplementary Figs 1 and 2). Clearly, values of *δ*_e_(*Y*^s^) lie on the lower tail of the null distribution (low quantile values) at more locations than they lie on the upper tail (for example, 23% of the analysed boxes lie below 0.1, while only 10% lie above 0.9 as expected by chance). This indicates that afternoon rain falls preferentially over soils that are drier than their surrounding consistently with the findings from ref. 11 (see also Supplementary Fig. 1a), with small differences in the regional patterns likely due to various factors (soil moisture data, precipitation data set version, months used for the analysis and so on). Hence, results from ref. 11 do not depend on the use of shallow surface soil moisture therein, as we reproduce these results when considering soil moisture over the entire root zone. The data set-related uncertainties are illustrated in Supplementary Figs 1 and 2, where various data set combinations are shown to lead to partly different patterns, while all agreeing on the strong dominance of negative spatial coupling. These uncertainties have little impact on our interpretation as we precisely focus on the overall emerging sign of the coupling rather than on its magnitude or geographical distribution.

### Temporal relationships

**Figure 2:**
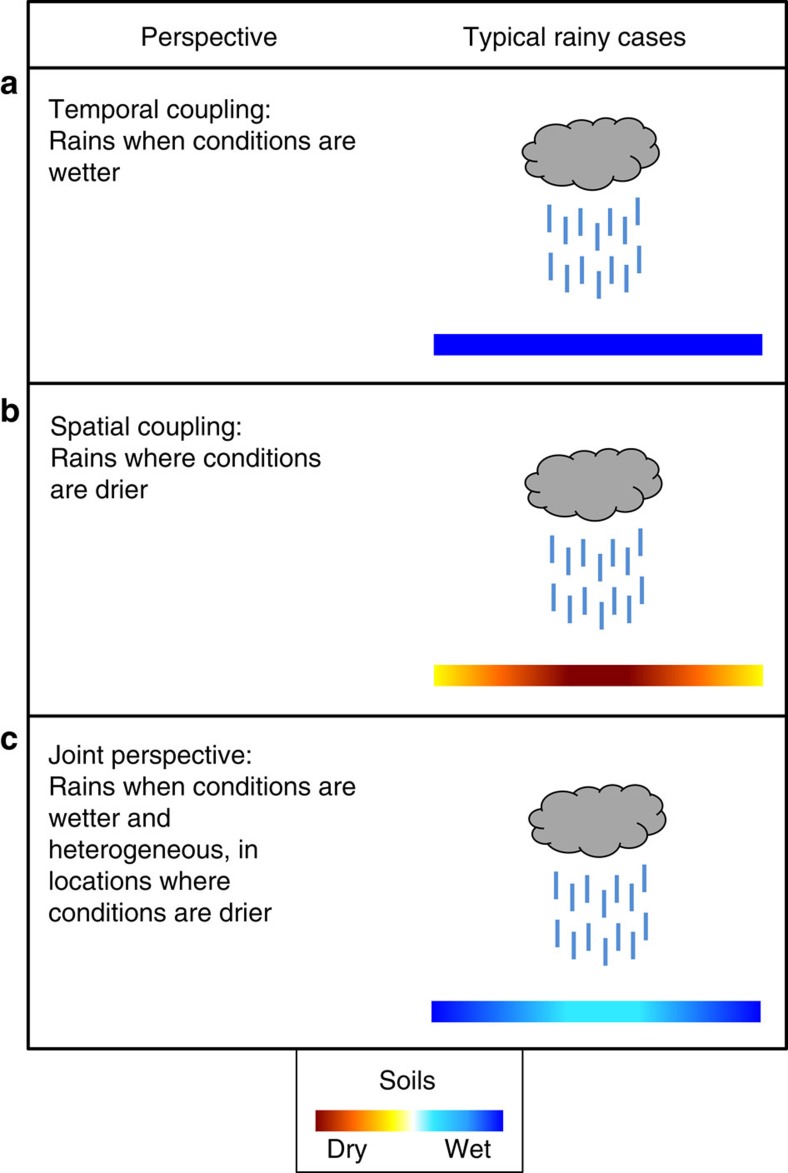
Representation of various perspectives on soil moisture–precipitation coupling. Traditionally in the literature, temporal approaches (**a**) suggest that rain is more likely in wet conditions, while spatial approaches (**b**) emphasize rain over locally drier patches. The joint perspective presented here (**c**) highlights that both are valid, and thereby rain is more likely in overall wet conditions but is located over drier (less wet) patches. Shown here are typical soil moisture conditions preceding afternoon rainfall events but do not necessarily imply causal relationships.

The temporal analysis based on *Y*^t^ provides information about the soil moisture state on the morning of an event compared with the expectation, by computing 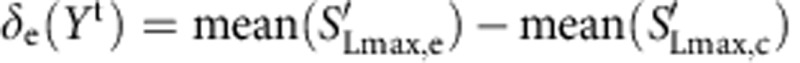. While this approach is likely to be impacted by externally forced precipitation persistence at various time scales^15^, it provides a temporal perspective that complements the spatial approach inherent to the metric by ref. 11. We find that afternoon rain does not occur preferentially on days with drier soil conditions: Fig. 1b (see also Supplementary Figs 3 and 4) highlights that the analysed precipitation events occur for most locations when soils are wetter than usual at Lmax (that is, positive temporal relationship; 51% of analysed grid boxes above 0.9 versus 10% below 0.1). Exceptions are the Central United States, Western Amazonia and parts of the Sahel and Equatorial Africa, where precipitation events tend to occur when soils are dry. We find similar results using Levt or Lmin instead of Lmax (Supplementary Figs 5 and 6; Supplementary Discussion), indicating that this correlation may be driven by soil moisture on a larger scale. Together, the spatial and temporal analyses highlight that, for most regions, precipitation events generally occur when soils are wet, but where soils are drier relative to larger-scale regions (as illustrated in Fig. 2).

### Possible mechanisms

One possible explanation for the diagnosed temporal relationships^14^,^15^ is that the atmosphere can sustain persistent large-scale features that favour sequences of dry (or wet) days, regardless of soil moisture state. On the other hand, if the above relationships indicate causality, our results imply that soil moisture–precipitation coupling interacts in two ways: the positive temporal coupling might enhance precipitation persistence, while the negative spatial coupling leads to an homogenization of moisture on land. However, these two simultaneous processes are likely to be interdependent, first of all because the required soil moisture heterogeneity for the presence of a spatial coupling may be temporally related to precipitation. To assess whether precipitation events indeed present a preference for heterogeneous soil moisture conditions, we compute our third metric based on *Y*^h^ and thereby compare spatial heterogeneity in the morning of event versus non-event days. The results (Fig. 1c; Supplementary Figs 7 and 8) clearly indicate that precipitation is triggered preferentially over heterogeneous soil moisture conditions.

Taken together, our results suggest that—if our metrics do not only reflect atmospheric persistence—the negative spatial coupling could lead to a positive temporal feedback at a larger scale, as precipitation-induced soil moisture heterogeneity might help generating further precipitation events via spatial coupling mechanisms^5^, although with a spatial shift related to previous events. In addition, the rain over drier parcels might enhance evaporation in more water-stressed patches, thereby increasing total evaporation over a larger region. These two effects, in turn could contribute to a positive temporal feedback at larger scales. Note that a positive temporal coupling would not need to occur locally but could also affect areas downwind via moisture recycling^16^. This view is also consistent with the fact that land evaporation is overall an important source of moisture for precipitation on land^1^,^16^. Nonetheless, a negative temporal effect might also be generated by spatial coupling, which tends to homogenize land wetness and thereby might reduce the occurrence of heterogeneity-induced precipitation events. These possible mechanisms are consistent with our results; their existence and relevance, however, depend on the relative contributions of soil moisture versus atmospheric persistence to the computed statistical relationships.

## Discussion

Our findings potentially reconcile a number of studies on soil moisture–precipitation feedback, as illustrated in Fig. 2. Indeed, we demonstrate the compatibility of a positive temporal correlation^8^,^10^,^15^,^17^,^18^ with a negative spatial correlation^5^,^11^. We show that the apparent contradiction in the sign of the soil moisture–precipitation coupling as estimated from various studies is not related to the underlying data but to the consideration of different aspects of the relationship between soil moisture and precipitation. These different aspects might be investigated by combining spatial and temporal metrics, which appear to be conceptually compatible (Fig. 2). Nevertheless, we underline that the apparent positive temporal coupling could reflect persistence in large-scale controls such as atmospheric moisture advection^14^,^15^. In such cases, it remains unclear whether a positive soil moisture–precipitation feedback contributes to precipitation persistence and the observed positive temporal relationship, or whether a weak or negative temporal coupling is hidden behind the atmospheric persistence.

Spatial investigations of the coupling present the advantage of directly addressing persistence, as nearby locations are likely to exhibit similar conditions on the same day. However, such approaches by definition relate to spatial heterogeneity and it is not clear if they fully address the question of whether a feedback occurs temporally^19^, which might be most relevant for seasonal forecasting^20^. Our temporal analysis of heterogeneity highlights potential interactions between spatial and temporal coupling mechanisms. Nonetheless, exhaustively addressing causality in temporal soil moisture–precipitation feedback has proven challenging over the past^1^,^14^,^15^,^21^ and will likely remain so, given the large discrepancies in soil moisture–precipitation coupling in climate models^2^ and their reported failure to capture some emergent features such as the discussed spatial soil moisture–precipitation relationship^11^. In any case, our results suggest that positive temporal feedback might more likely occur at larger (regional) scales rather than be related to boundary-layer dynamics at the local scale (at least not in comparison with surrounding pixels).

In spite of these open questions, we demonstrate that the definition of soil moisture–precipitation coupling in a temporal or spatial context plays a crucial role in the resulting sign of the relationship. Temporally, we find the dominance of an apparent positive relationship, that is, rain occuring more often in wet conditions, which might enhance precipitation persistence. Spatially, we find a dominant negative relationship, that is, rain occuring more often over soils that are drier than the surrounding areas, which might lead to an homogenization of moisture availability on land. If representative of causal relationships, these results would be consistent with the notion of moisture recycling^3^, as well as the existence of soil moisture-induced mesoscale circulations^5^. Improvements in models, in particular with respect to the representation of convection, as well as studies of the feedback with models that explicitly resolve convection^13^, are becoming increasingly crucial to help disentangling the observed positive temporal relationship from atmospheric persistence.

## Methods

### Precipitation data

We use three precipitation data sets that merge measurements from a number of satellites to produce quasi-global, consistent data sets at a high spatial (0.25° × 0.25°) and temporal (3 h) resolution: CMORPH (the Climate Prediction Center morphing method^22^) and PERSIANN (Precipitation Estimation from Remotely Sensed Information using Artificial Neural Networks^23^), available from 60° S to 60° N, and TRMM3B42 (from the Tropical Rainfall Measuring Mission^24^), available from 50° S to 50° N and hereafter referred to as TRMM. These products provide us with at least partly independent databases for our investigation of land–precipitation coupling. Here we choose CMORPH as our main data set because of the more physically based algorithm employed, and we only show results with this data set in Fig. 1. Results with other precipitation data sets are provided in the Supplementary Information (Supplementary Figs 1–8).

All three products are primarily based on data from passive microwave sensor overpasses, which provide high-quality precipitation estimates, but are available typically only several times a day. These are combined with data from infrared sensors onboard geostationary satellites, which are available at a high temporal resolution over most of the globe. The products rely on different algorithms to convert the raw measurements to consistent precipitation data.

CMORPH propagates passive estimates using motion vectors derived from infrared sensors^22^. We use version 1.0 of CMORPH, where the whole archive was reprocessed using a fixed algorithm and using inputs of the same versions. TRMM uses data from the passive microwave sensors, incorporates radars on the TRMM satellites and fills the gaps with infrared data calibrated at the monthly time scale before scaling estimates to monthly rain gauge observations^24^. We use version 7 of the 3B42 product. PERSIANN is based on roughly the same input satellite data but uses a neural network approach to estimate precipitation^23^.

These products have been validated and used in numerous studies^25^,^26^,^27^. Generally, a good agreement is found with other products^28^, but the quality decreases at high latitudes and over water bodies, complex terrain or coastal areas^29^. Nonetheless, our analysis does not consider areas with complex topography and water bodies. In addition, precipitation occurrence, which is at the basis of our precipitation detection analysis, was shown to be of good quality^30^. Before conducting our analyses, the 3-h precipitation data was adjusted to local time (based on longitude) by taking the closest 3-h Coordinated Universal Time-based time step.

### Soil moisture data

We use two main soil moisture data sets based on satellite observations: surface soil moisture from AMSR-E (NASA-LPRM algorithm^31^) and total evaporative stress from GLEAM (‘Global Land Evaporation: the Amsterdam Methodology’^32^). We choose GLEAM as our primary data set, used in Fig. 1, because of two major advantages over satellite-based surface soil moisture estimates: it includes the whole root zone (in addition to surface soil moisture; limited to the top few cm for AMSR-E), and its variability is limited to when soil moisture impacts evaporation. It also accounts for the effect of the development of vegetation on the evaporative stress, via the inclusion of vegetation optical depth^32^. Its main drawback is that it relies on a process-based model to combine relevant observations. Note that the GLEAM data set used here assimilates AMSR-E data, and thereby the analysis is restricted to the AMSR-E era (2002–2011).

GLEAM estimates daily evaporative stress and evaporation components based on remote-sensing data of radiation, precipitation, air temperature, soil moisture, vegetation optical depth and snow water equivalents (see Supplementary Tables 1 and 2 for input data). Here we use a modified version of GLEAM^15^, which provides estimates of morning (09:00) total evaporative stress *S*, defined by *E*=*SE*_pot_, where *E* denotes evaporation from the land surface and *E*_pot_ is potential evaporation, estimated from the Priestley and Taylor approach^33^ in GLEAM. Thus, *S* quantifies the land surface stress on evaporation from soil moisture and vegetation activity, and it is referred to as ‘soil moisture stress’, as the impacts of vegetation on *S* are occurring over slower time scales than the one analysed here.

For each land fraction *i*, the evaporative stress in GLEAM (*S*_*i*_) is defined as a linear function between the wilting point (soil moisture level below which no water is available to plant, that is, *S*_i_=0) and a critical soil moisture level (for and above which *S*_*i*_=*S*_*i*,max_, where *S*_*i*,max_ is a function of vegetation optical depth). For the bare soil fraction, *S*_s,max_=1. Where not stated otherwise, we use the total stress *S* defined as the area-weighted average of individual *S*_*i*_ values over bare soils, short and tall vegetation.

The original GLEAM formulation provides estimates at daily time steps. We adapt this formulation to match our specific timing requirements and thus obtain estimates at 09:00. To do so, we drive GLEAM with input data shown in Supplementary Table 1 and by aggregating all variables to a daily cycle starting and ending at 09:00, similarly to the procedure described in ref. 15. *S* does not include the effect of vegetation interception, but the presence of intercepted water in the morning is unlikely as days with morning rain are removed from the analysis^15^ (see below).

To test the sensitivity of our results to the precipitation data used as input, we compute three estimates from our three precipitation data sets, which we refer to as GLEAM_C_, GLEAM_T_ and GLEAM_P_ for the estimates driven by CMORPH, TRMM and PERSIANN, respectively. Results shown in Fig. 1 refer to GLEAM_C_.

A major difference in our input data sets compared with ref. 15 is the use of surface radiation data from CERES (the Clouds and Earth’s Radiation Energy System^34^) instead of the GEWEX Surface Radiation Balance data^35^. These two data sets are based on multiple satellite data and they provide top-of-the-atmosphere and surface radiation fluxes globally, at a high temporal resolution (3 h)^36^. CERES is based on more recent sensors, with data starting and extending later in time, which provides longer overlap with the other products used in the analysis (CMORPH and PERSIANN in particular). In addition, CERES surface products have been shown to perform well^37^,^38^,^39^.

Data gaps are filled using GPCP (for precipitation) and ERA-interim (for radiation, and for precipitation in cases where GPCP data are missing) as shown in Supplementary Table 2. All data sets are interpolated bilinearly to a 0.25° resolution before the pre-processing. GPCP is a daily product. For each day, gaps in a 3 h time step in CMORPH or PERSIANN are filled to match daily precipitation from GPCP. Other data sets are available at 3 h resolution. To mitigate the impacts of gap filling, GLEAM 09:00 values of day *i* are masked and removed from the analysis if precipitation or net radiation is missing on day *i*−1, or if in the 10 previous days, a gap of *n* subsequent days in precipitation data ends between day *i*−*n*/2 and day *i*−1.

GLEAM has been extensively validated and inter-compared with other methodologies to estimate heat fluxes^32^,^40^,^41^,^42^,^43^. In particular, soil moisture from GLEAM has been successfully validated using measurements from 701 soil moisture sensors all across the world (see Supplementary Information of ref. 44). The mean and s.d. of GLEAM *S* are shown in Supplementary Fig. 9. The sensitivity of our results to the chosen soil moisture data set has been investigated by using not only satellite-based surface soil moisture from AMSR-E (Supplementary Figs 1,3 and 5–7) but also GLEAM estimates based on various precipitation inputs (Supplementary Figs 1,3 and 5–7) and surface soil moisture stress from GLEAM (Supplementary Figs 2, 4 and 8). Note that the forcing data, especially the surface soil moisture observations, are prone to large errors over tropical forests. Results in these regions should therefore be interpreted with caution.

### Computational details

We use the precipitation event detection technique from ref. 11 and compare the pre-event soil moisture field of the events with a control sample based on non-event days. We define afternoon precipitation as the accumulated precipitation between 12:00 and 24:00. On a particular day, an event consists of 5 × 5 grid cells (0.25° × 0.25° each, that is, 1.25° × 1.25° in total) centred at a location of local maxima (Lmax) where afternoon precipitation exceeds 4 mm. In case of overlap between several events on the same day, only the event with the largest accumulated precipitation at Lmax is retained. The 1.25° × 1.25° event domain is denoted as Levt, while Lmin is the location of precipitation minimum within Levt, where in case of multiple grid cells belonging to Lmin for a given event, the soil moisture value at Lmin is defined as the average of all the corresponding values.

A number of filters are applied to the individual 0.25° grid cells, and events for which Levt contains any filtered out grid cell are excluded from the computation. To ensure that events are generated in the analysed afternoon, grid cells with morning (06:00−12:00) precipitation >1 mm are filtered out. Grid cells with fixed features that may influence the precipitation field are also excluded: grid cells with a range of topographic height within a box of 1.25° exceeding 300 m, as well as grid cells where water bodies cover over 5% of the area are removed as in ref. 11, using the data sets therein. Supplementary Figure 10 illustrates the event definition and the filters applied with an example day over West Africa. To concentrate on the convective season, results in Fig. 1 are presented for May–September latitudes North of 23° N, November–March latitudes South of 23° S, and including all months in the tropics where convection is the dominant process of precipitation generation^45^. Results for individual seasons are available in Supplementary Fig. 11.

Once the events are detected, we analyse the corresponding patterns of morning soil moisture defined by *Y* (that is, *Y*^s^, *Y*^t^ or *Y*^h^, see main text). Soil moisture anomalies *S*′ (that is, with the mean seasonal cycle subtracted) are used to mitigate the impacts of seasonality. The control sample corresponding to each event includes all days from the same calendar month on different years, excluding days with morning rainfall or with an event occurring at the same location. Before pooling events belonging to a fixed 5° box together, the climatological value of *Y* is subtracted to ensure comparability between locations. In total, a large number of events is detected for most boxes, in particular over tropical areas (Supplementary Fig. 12).

Typical *δ*(*Y*) values are computed by pooling both samples (*Y*_e_ and *Y*_c_) together and taking 1,000 bootstrap samples of a size equal to the size of *Y*_e_. The quantile of the null distribution of *δ*(*Y*) to which the actual *δ*_e_(*Y*) corresponds is a measure of the significance of the relationship.

Note that we have chosen to include rainfall from 12:00 to 24:00 in our analysis, while ref. 11 uses 12:00−21:00. This might lead to some different detected locations if events are propagated. However, a direct comparison between the two definitions of afternoon shown in Supplementary Fig. 13 highlights the robustness of our results to this aspect of the analysis.

Applying a temporal metric from the literature^10^,^15^ to our data yields regions of positive and negative *P* values similar to our temporal metric *δ*_e_(*Y*^t^) shown in Fig. 1b (see Supplementary Discussion; Supplementary Figs 14 and 15).

## Author contributions

B.P.G., B.O. and S.I.S. initiated the study. B.P.G. mainly performed the analysis and wrote the manuscript. D.G.M. contributed to the analysis. All authors participated in the design of the experiments, discussion of the results and writing of the paper.

## Additional information

**How to cite this article:** Guillod, B. P. *et al*. Reconciling spatial and temporal soil moisture effects on afternoon rainfall. *Nat. Commun*. 6:6443 doi: 10.1038/ncomms7443 (2015).

## Supplementary Material

Supplementary Information Supplementary Figures 1-15, Supplementary Tables 1-2, Supplementary Discussion and Supplementary References

## References

[b1] Seneviratne S. I. . Investigating soil moisture-climate interactions in a changing climate: a review. Earth-Sci. Rev. 99, 125–161 (2010) .

[b2] Koster R. D. . Regions of strong coupling between soil moisture and precipitation. Science 305, 1138–1140 (2004) . 15326351 10.1126/science.1100217

[b3] Eltahir E. A. B. & Bras R. L. Precipitation recycling. Rev. Geophys. 34, 367–378 (1996) .

[b4] Ek M. B. & Holtslag A. A. M. Influence of soil moisture on boundary layer cloud development. J. Hydrometeorol. 5, 86–99 (2004) .

[b5] Taylor C. M. . Frequency of Sahelian storm initiation enhanced over mesoscale soil-moisture patterns. Nat. Geosci 4, 1–4 (2011) .

[b6] Findell K. L. & Eltahir E. A. B. Atmospheric controls on soil moisture-boundary layer interactions. Part I: framework development. J. Hydrometeorol. 4, 552–569 (2003) .

[b7] Gentine P., Holtslag A. A. M., D'Andrea F. & Ek M. Surface and atmospheric controls on the onset of moist convection over land. J. Hydrometeorol. 14, 1443–1462 (2013) .

[b8] Koster R. D., Suarez M. J., Higgins R. W. & Van den Dool H. M. Observational evidence that soil moisture variations affect precipitation. Geophys. Res. Lett. 30, 1–4 (2003) .

[b9] Guo Z. . GLACE: the global land-atmosphere coupling experiment. Part II: analysis. J. Hydrometeorol. 7, 611–625 (2006) .

[b10] Findell K. L., Gentine P., Lintner B. R. & Kerr C. Probability of afternoon precipitation in eastern United States and Mexico enhanced by high evaporation. Nat. Geosci. 4, 434–439 (2011) .

[b11] Taylor C. M., de Jeu R. A. M., Guichard F., Harris P. P. & Dorigo W. A. Afternoon rain more likely over drier soils. Nature 489, 423–426 (2012) . 22972193 10.1038/nature11377

[b12] Hohenegger C., Brockhaus P., Bretherton C. S. & Schär C. the soil moisture-precipitation feedback in simulations with explicit and parameterized convection. J. Clim. 22, 5003–5020 (2009) .

[b13] Taylor C. M. . Modeling soil moisture-precipitation feedback in the Sahel: Importance of spatial scale versus convective parameterization. Geophys. Res. Lett. 40, 6213–6218 (2013) .

[b14] Salvucci G. D., Saleem J. A. & Kaufmann R. Investigating soil moisture feedbacks on precipitation with tests of Granger causality. Adv. Water Resour. 25, 1305–1312 (2002) .

[b15] Guillod B. P. . Land-surface controls on afternoon precipitation diagnosed from observational data: uncertainties and confounding factors. Atmos. Chem. Phys. 14, 8343–8367 (2014) .

[b16] van der Ent R. J. & Savenije H. H. G. Length and time scales of atmospheric moisture recycling. Atmos. Chem. Phys. 11, 1853–1863 (2011) .

[b17] Alfieri L., Claps P., D'Odorico P., Laio F. & Over T. M. An Analysis of the soil moisture feedback on convective and stratiform precipitation. J. Hydrometeorol. 9, 280–291 (2008) .

[b18] Ferguson C. R., Wood E. F. & Vinukollu R. K. A Global Intercomparison of Modeled and Observed Land-Atmosphere Coupling. J. Hydrometeorol. 13, 749–784 (2012) .

[b19] Koster R. D. Climate science: storm instigation from below. Nat. Geosci. 4, 427–428 (2011) .

[b20] Koster R. D. . Contribution of land surface initialization to subseasonal forecast skill: First results from a multi-model experiment. Geophys. Res. Lett. 37, 1–6 (2010) .

[b21] Teuling A. J., Uijlenhoet R. & Troch P. A. On bimodality in warm season soil moisture observations. Geophys. Res. Lett. 32, 10–13 (2005) .

[b22] Joyce R. J., Janowiak J. E., Arkin P. A. & Xie P. CMORPH: a method that produces global precipitation estimates from passive microwave and infrared data at high spatial and temporal resolution. J. Hydrometeorol. 5, 487–503 (2004) .

[b23] Hsu K.-l., Gao X., Sorooshian S. & Gupta H. V. Precipitation estimation from remotely sensed information using artificial neural networks. J. Appl. Meteorol. 36, 1176–1190 (1997) .

[b24] Huffman G. J. . The TRMM multisatellite precipitation analysis (TMPA): quasi-global, multiyear, combined-sensor precipitation estimates at fine scales. J. Hydrometeorol. 8, 38–55 (2007) .

[b25] Sorooshian S. . Evaluation of PERSIANN system satellite-based estimates of tropical rainfall. Bull. Am. Meteorol. Soc. 81, 2035–2046 (2000) .

[b26] Habib E., Haile A. T., Tian Y. & Joyce R. J. Evaluation of the high-resolution CMORPH satellite rainfall product using dense rain gauge observations and radar-based estimates. J. Hydrometeorol. 13, 1784–1798 (2012) .

[b27] Dai A., Lin X. & Hsu K.-L. The frequency, intensity, and diurnal cycle of precipitation in surface and satellite observations over low- and mid-latitudes. Clim. Dyn 29, 727–744 (2007) .

[b28] Tian Y. & Peters-Lidard C. D. A global map of uncertainties in satellite-based precipitation measurements. Geophys. Res. Lett. 37, L24407 (2010) .

[b29] Tian Y. & Peters-Lidard C. D. Systematic anomalies over inland water bodies in satellite-based precipitation estimates. Geophys. Res. Lett. 34, L14403 (2007) .

[b30] Tian Y. . Component analysis of errors in satellite-based precipitation estimates. J. Geophys. Res. 114, D24101 (2009) .

[b31] Owe M., de Jeu R. & Holmes T. Multisensor historical climatology of satellite-derived global land surface moisture. J. Geophys. Res. 113, F01002 (2008) .

[b32] Miralles D. G. . Global land-surface evaporation estimated from satellite-based observations. Hydrol. Earth Syst. Sci. 15, 453–469 (2011) .

[b33] Priestley C. H. B. & Taylor R. J. On the assessment of surface heat flux and evaporation using large-scale parameters. Mon. Wea. Rev. 100, 81–92 (1972) .

[b34] Wielicki B. A. . Clouds and the Earth's radiant energy system (CERES): an earth observing system experiment. Bull. Am. Meteorol. Soc. 77, 853–868 (1996) .

[b35] Stackhouse P. W. . 12 year surface radiation budget data set. GEWEX News 14, 10–12 (2004) .

[b36] Young D. F., Minnis P., Doelling D. R., Gibson G. G. & Wong T. Temporal Interpolation Methods for the Clouds and the Earth's Radiant Energy System (CERES) Experiment. J. Appl. Meteorol. 37, 572–590 (1998) .

[b37] Kato S. . Improvements of top-of-atmosphere and surface irradiance computations with CALIPSO-, CloudSat-, and MODIS-derived cloud and aerosol properties. J. Geophys. Res. 116, D19209 (2011) .

[b38] Kato S. . Uncertainty estimate of surface irradiances computed with MODIS-, CALIPSO-, and CloudSat-derived cloud and aerosol properties. Surv. Geophys. 33, 395–412 (2012) .

[b39] Kato S. . Surface irradiances consistent with ceres-derived top-of-atmosphere shortwave and longwave irradiances. J. Clim. 26, 2719–2740 (2013) .

[b40] Miralles D. G., De Jeu R. A. M., Gash J. H., Holmes T. R. H. & Dolman A. J. Magnitude and variability of land evaporation and its components at the global scale. Hydrol. Earth Syst. Sci. 15, 967–981 (2011) .

[b41] Liu Y. . Response of evapotranspiration and water availability to changing climate and land cover on the Mongolian Plateau during the 21st century. Glob. Planet. Chang. 108, 85–99 (2013) .

[b42] Mueller B. . Benchmark products for land evapotranspiration: LandFlux-EVAL multi-data set synthesis. Hydrol. Earth Syst. Sci. 17, 3707–3720 (2013) .

[b43] Trambauer P. . Comparison of different evaporation estimates over the African continent. Hydrol. Earth Syst. Sci. 18, 193–212 (2014) .

[b44] Miralles D. G. . El Niño-La Niña cycle and recent trends in continental evaporation. Nat. Clim. Chang. 4, 122–126 (2014) .

[b45] Yang S. & Smith E. A. Convective-stratiform precipitation variability at seasonal scale from 8 yr of trmm observations: implications for multiple modes of diurnal variability. J. Clim. 21, 4087–4114 (2008) .

