# Supplementary material for: Reconciling spatial and temporal soil moisture effects on afternoon rainfall
Source: Nat Commun. 2015 Mar 5;6:6443. doi: 10.1038/ncomms7443 (PMC4366536; doi:10.1038/ncomms7443)
Supplement: Supplementary Information — Supplementary Figures 1-15, Supplementary Tables 1-2, Supplementary Discussion and Supplementary References [file ncomms7443-s1.pdf]

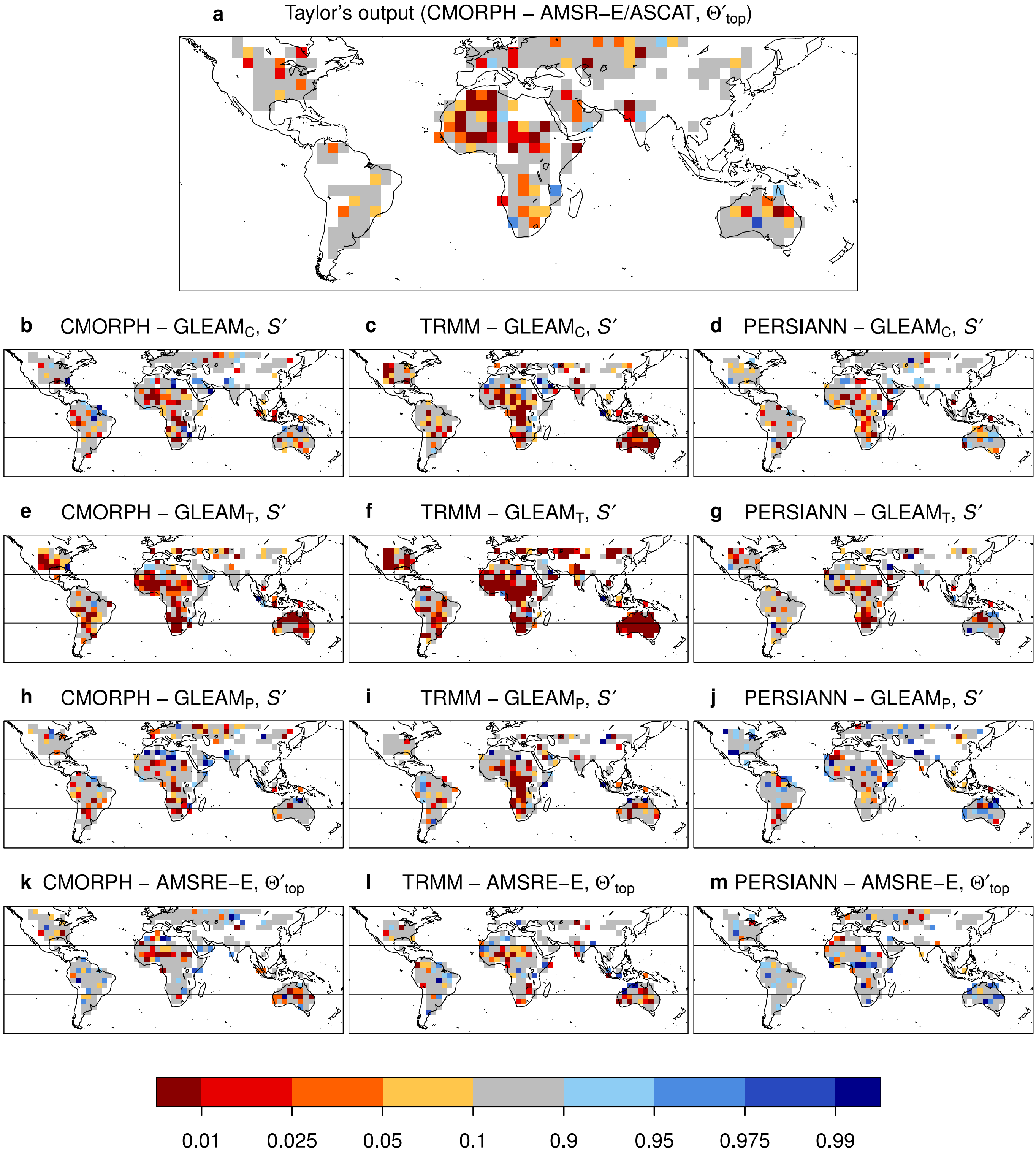

Supplementary Figure 1 | Spatial results (as for Fig. 1a) for multiple soil moisture (rows) and precipitation (columns) datasets. Quantile of $\delta_{\mathrm{e}}(Y^{\mathrm{s}})$ where $Y^{\mathrm{s}} = \Delta X' = X'_{\mathrm{Lmax}} - X'_{\mathrm{Lmin}}$ and X' are anomalies of (**a**) surface soil moisture (Θ_{top}) merged from AMSR-E and ASCAT (results from ref. 1), (**b-j**) total soil moisture stress (S) from GLEAM with different input precipitation data, (**k-m**) surface soil moisture (Θ_{top}) from AMSR-E. Horizontal black lines (b-m) indicate the latitudes at which different months are included in the analysis (see Methods), while all months are included for all latitudes in (a). Boxes with at least 25 events are displayed and grey shading indicates non-significant relationships. Note that for panel (a) version 0 of CMORPH was used.

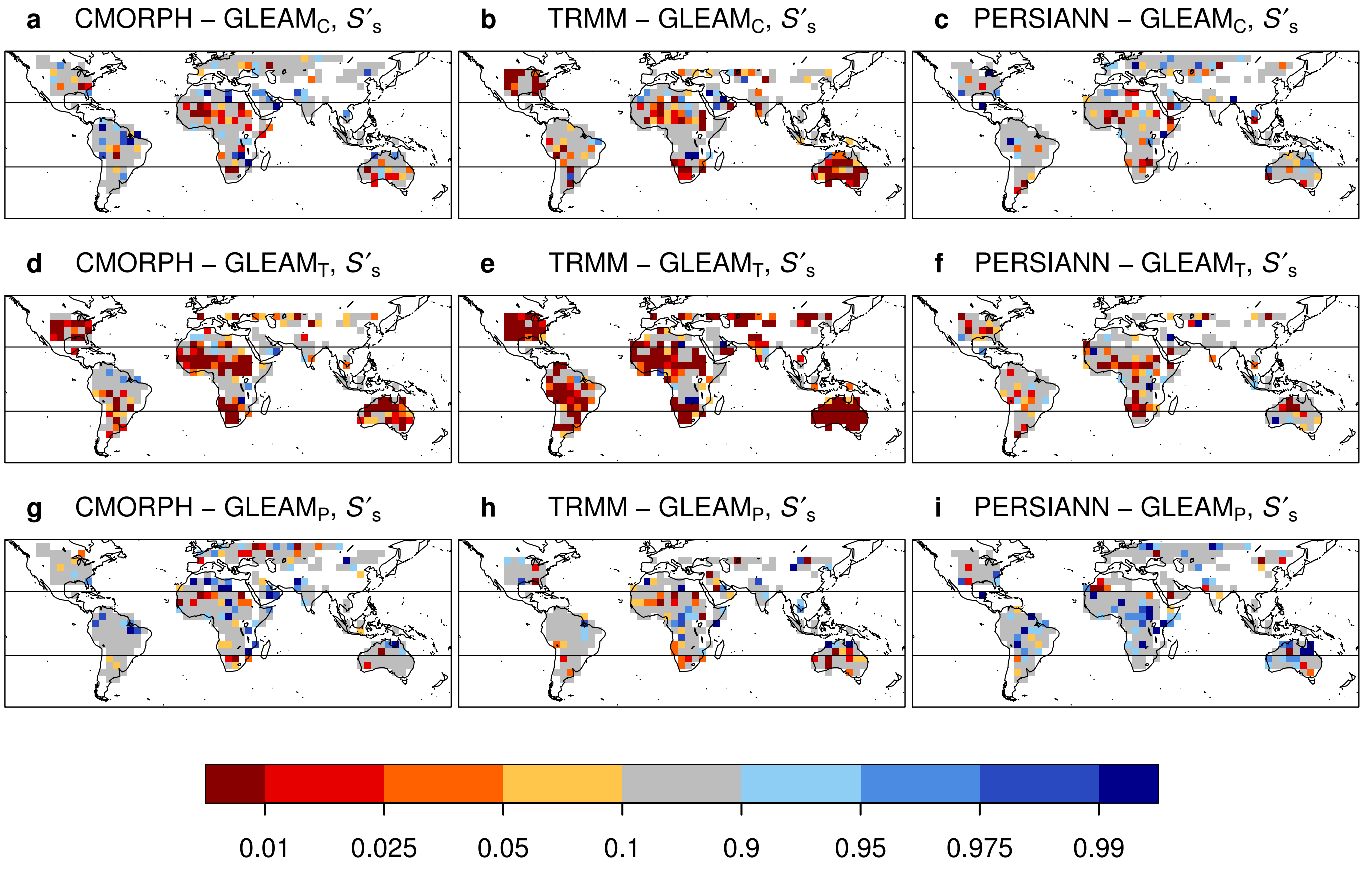

Supplementary Figure 2 | Spatial results (as for Fig. 1a and Supplementary Fig. 1) for multiple precipitation datasets (columns) and for surface soil moisture stress from GLEAM derived with multiple precipitation datasets (rows). Quantile of $\delta_e(Y^s)$ where $Y^s = \Delta S'_s = X'_{\mathrm{Lmax}} - X'_{\mathrm{Lmin}}$ and X' are anomalies of surface soil moisture stress from GLEAM ($X = S_s$). Horizontal black lines indicate the latitudes at which different months are included in the analysis (see Methods). Boxes with at least 25 events are displayed and grey shading indicates non-significant relationships.

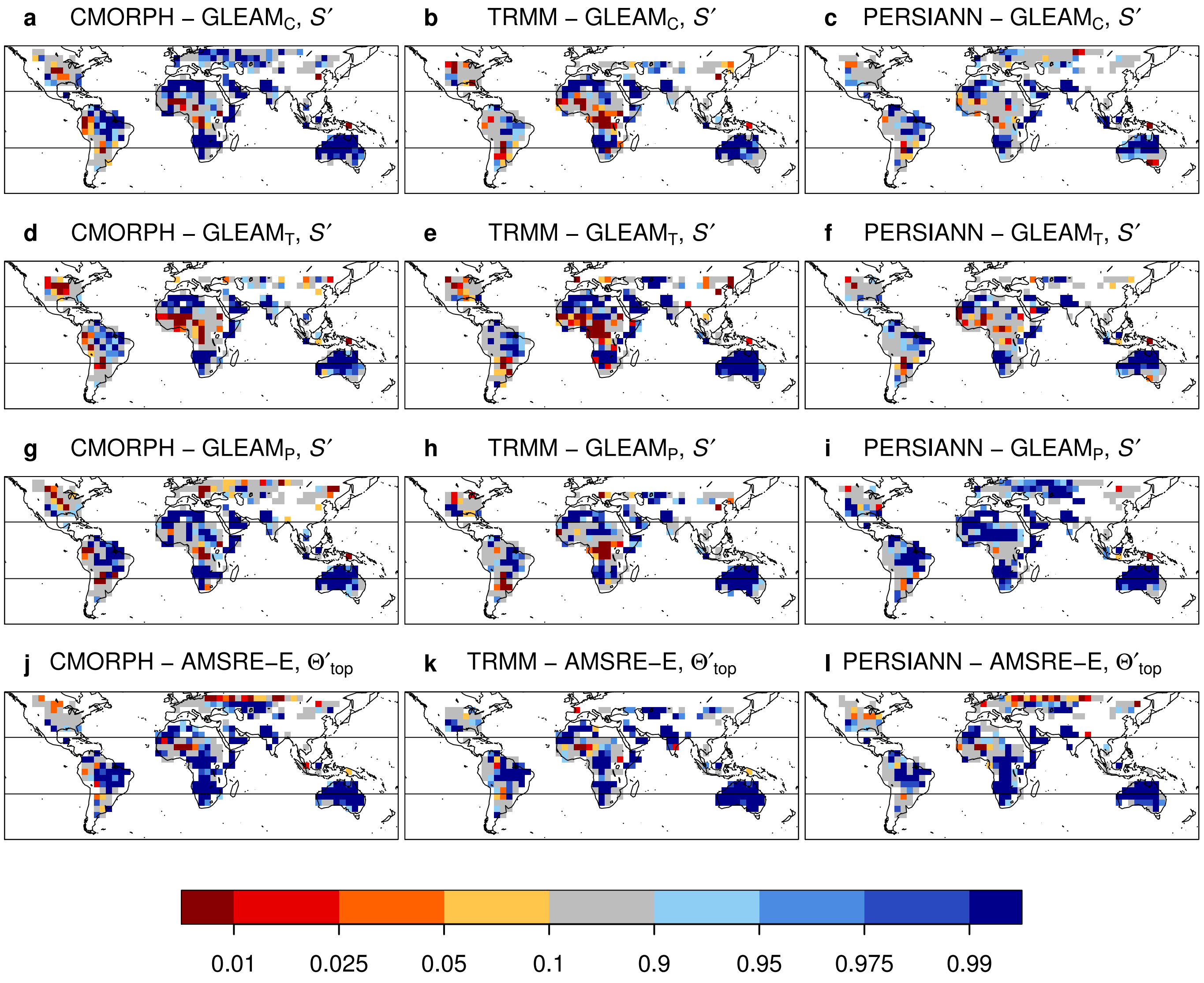

Supplementary Figure 3 | Temporal results (as for Fig. 1b) for multiple soil moisture (rows) and precipitation (columns) datasets. Quantile of $\delta_{\mathrm{e}}(Y^{\mathrm{t}})$ where $Y^{\mathrm{t}} = X'_{\mathrm{Lmax}}$ are anomalies, at Lmax, of (**a-i**) total soil moisture stress (S') from GLEAM with different input precipitation data, (**j-l**) surface soil moisture (Θ'_{top}) from AMSR-E. Horizontal black lines indicate the latitudes at which different months are included in the analysis (see Methods). Boxes with at least 25 events are displayed and grey shading indicates non-significant relationships.

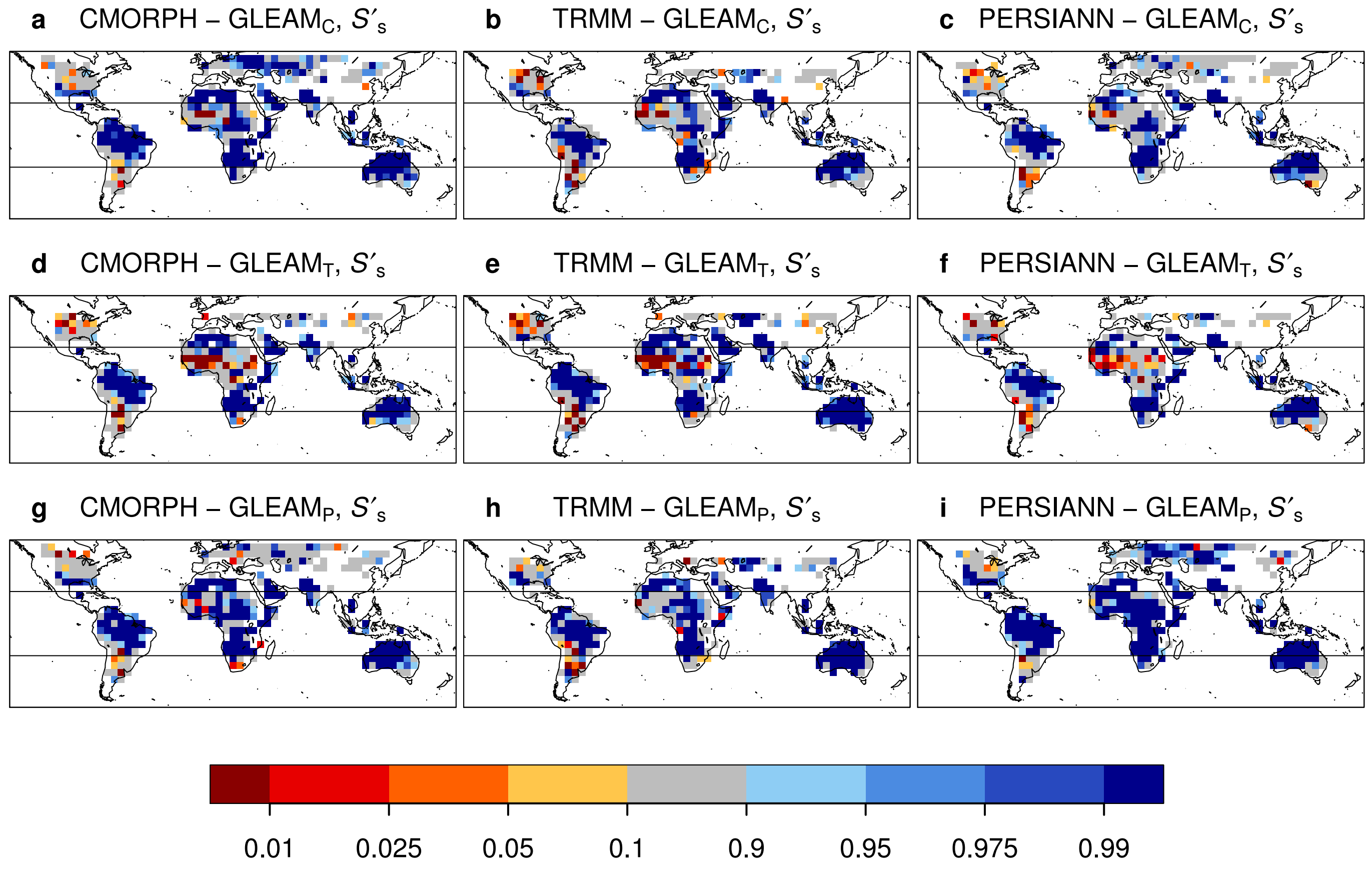

Supplementary Figure 4 | Temporal results (as for Fig. 1b and Supplementary Fig. 3) for multiple precipitation datasets (columns) and for surface soil moisture stress from GLEAM derived with multiple precipitation datasets (rows). Quantile of $\delta_{\mathrm{e}}(Y^{\mathrm{t}})$ where $Y^{\mathrm{t}} = X'_{\mathrm{Lmax}}$ are anomalies, at Lmax, of surface soil moisture stress ($X' = S'_{\mathrm{s}}$) from GLEAM. Horizontal black lines indicate the latitudes at which different months are included in the analysis (see Methods). Boxes with at least 25 events are displayed and grey shading indicates non-significant relationships.

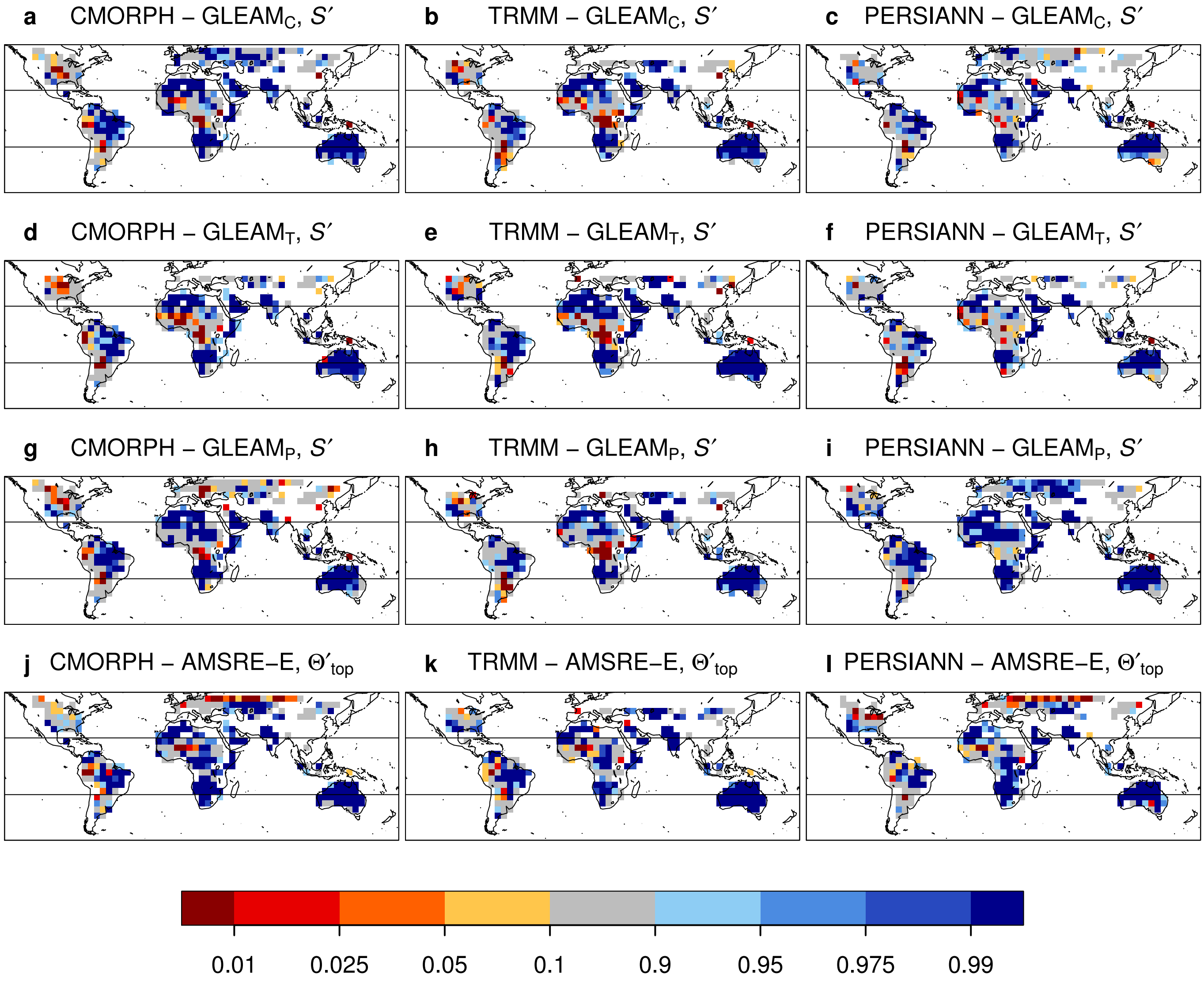

Supplementary Figure 5 | Temporal results using soil moisture from Levt (5 × 5 grid cells surrounding Lmax) instead of Lmax (Supplementary Fig. 3), for multiple soil moisture (rows) and precipitation (columns) datasets. Quantile of $\delta_e(X'_{\mathrm{Levt}})$ where X'_{Levt} are anomalies, at Levt, of (**a-i**) total soil moisture stress (S') from GLEAM with different input precipitation data, (**j-l**) surface soil moisture (Θ_{top}) from AMSR-E. Horizontal black lines indicate the latitudes at which different months are included in the analysis (see Methods). Boxes with at least 25 events are displayed and grey shading indicates non-significant relationships.

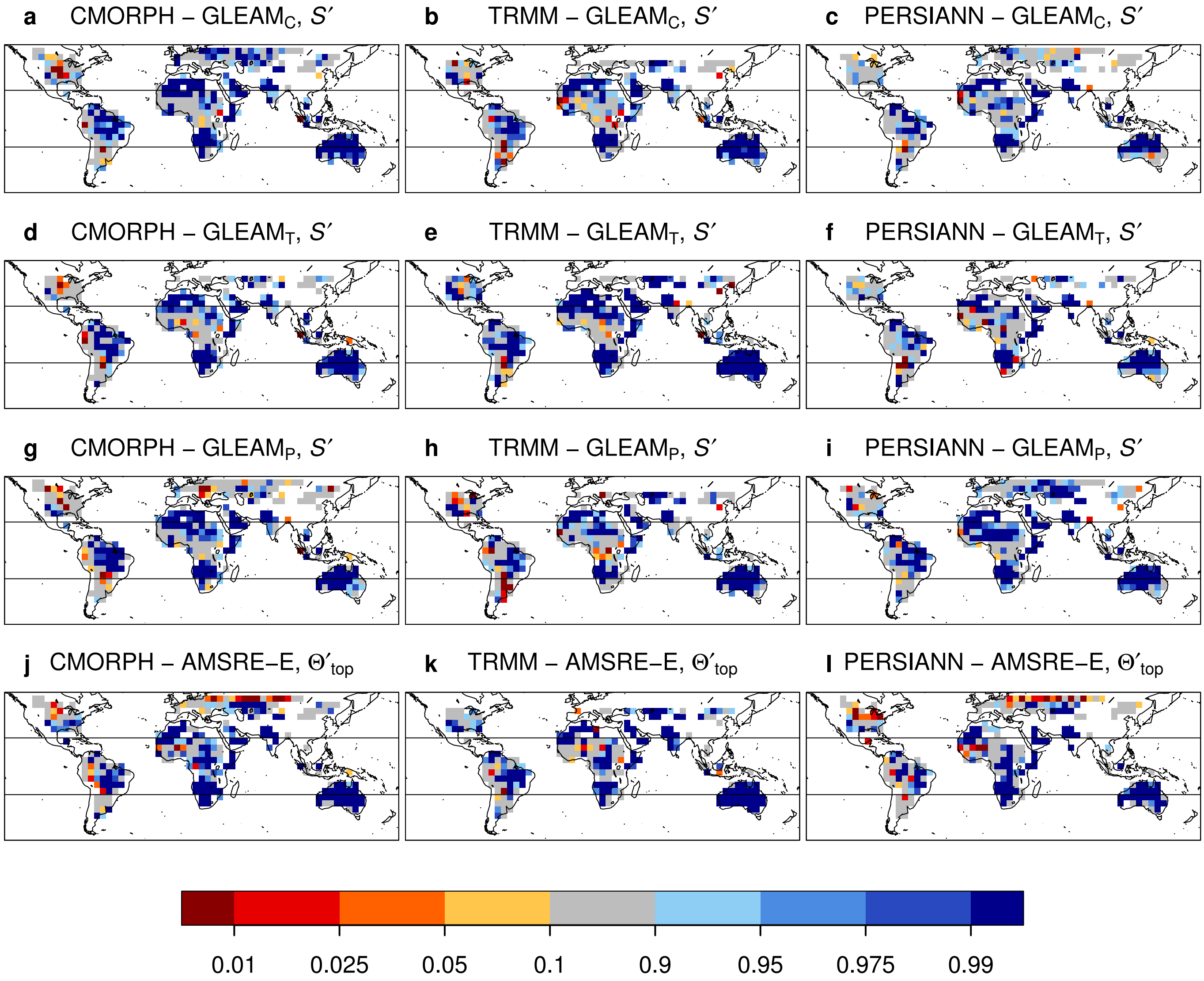

Supplementary Figure 6 | Temporal results using soil moisture from Lmin (location of rainfall minimum within Levt) instead of Lmax, for multiple soil moisture (rows) and precipitation (columns) datasets. Quantile of $\delta_e(X'_{\mathrm{Lmin}})$ where X'_{Lmin} are anomalies, at Lmin, of (**a-i**) total soil moisture stress (S') from GLEAM with different input precipitation data, (**j-l**) surface soil moisture (Θ'_{top}) from AMSR-E. Horizontal black lines indicate the latitudes at which different months are included in the analysis (see Methods). Boxes with at least 25 events are displayed and grey shading indicates non-significant relationships.

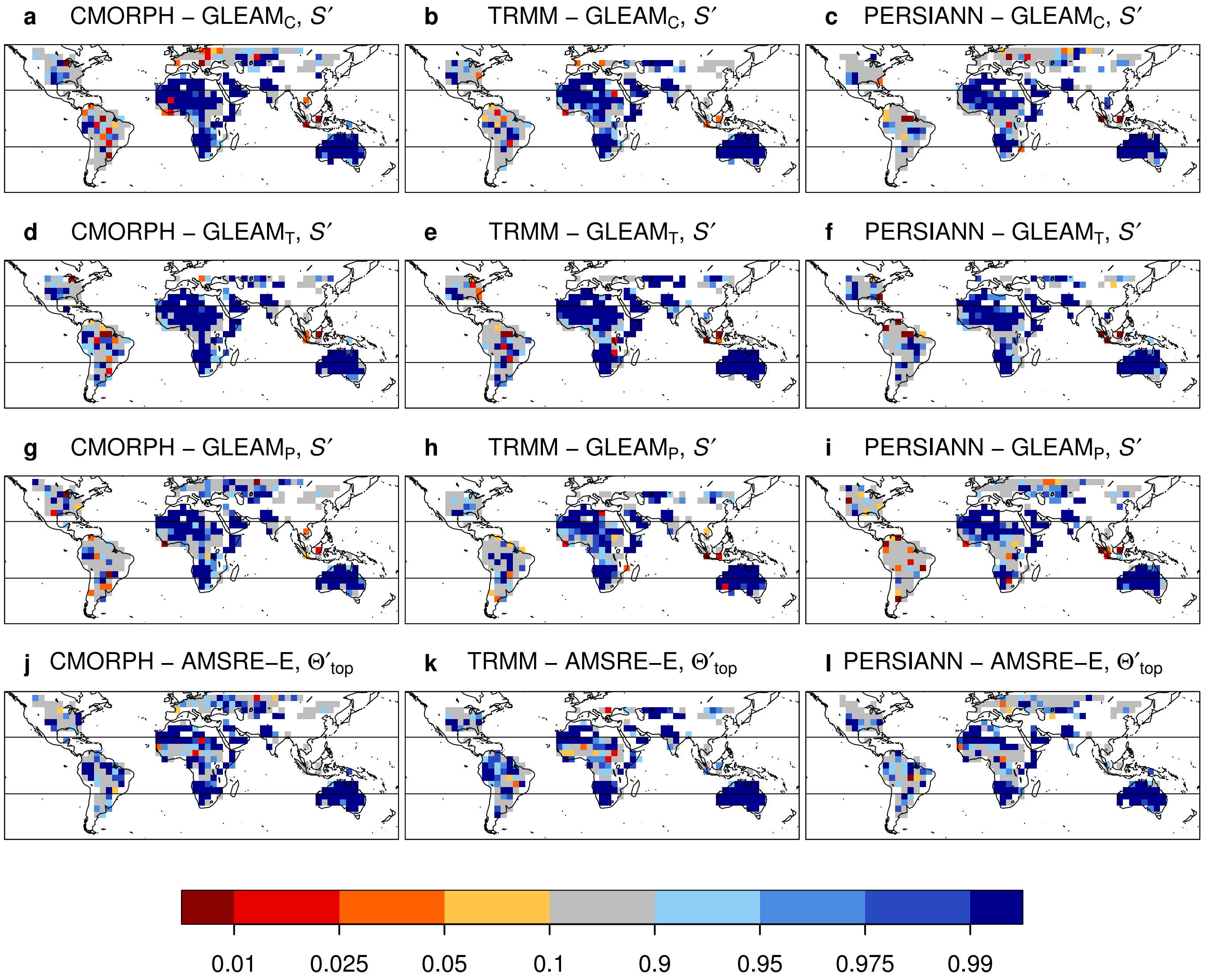

Supplementary Figure 7 | Temporal results using soil moisture heterogeneity (as for Fig. 1c), for multiple soil moisture (rows) and precipitation (columns) datasets. Quantile of $\delta_{\mathrm{e}}(Y^{\mathrm{h}})$ where $Y^{\mathrm{h}} = \sigma^{\mathrm{sp}}_{X'}$ is the spatial standard deviation, using the 25 grid cells within Levt, of X'. X' are anomalies of (**a-i**) total soil moisture stress (S') from GLEAM with different input precipitation data, (**j-l**) surface soil moisture (Θ'_{top}) from AMSR-E. Horizontal black lines indicate the latitudes at which different months are included in the analysis (see Methods). Boxes with at least 25 events are displayed and grey shading indicates non-significant relationships.

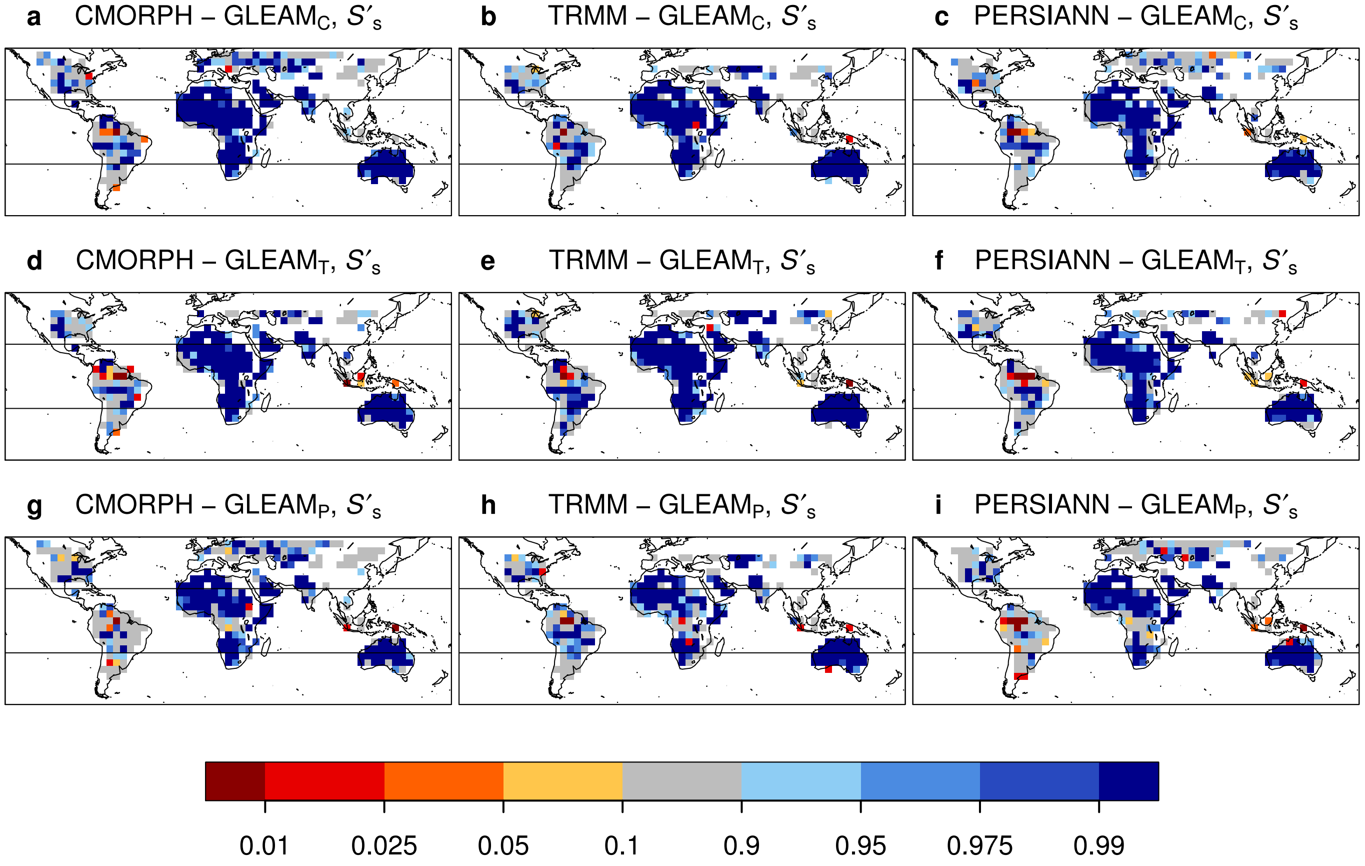

Supplementary Figure 8 | Temporal results using soil moisture heterogeneity (as for Fig. 1c and Supplementary Fig. 7) for multiple precipitation datasets (columns) and for surface soil moisture stress from GLEAM derived with multiple precipitation datasets (rows). Quantile of $\delta_{\mathrm{e}}(Y^{\mathrm{h}})$ where $Y^{\mathrm{h}} = \sigma^{\mathrm{sp}}_{X'}$ is the spatial standard deviation, using the 25 grid cells within Levt, of X'. X' are anomalies of surface soil moisture stress ($X' = S'_{\mathrm{s}}$) from GLEAM. Horizontal black lines indicate the latitudes at which different months are included in the analysis (see Methods). Boxes with at least 25 events are displayed and grey shading indicates non-significant relationships.

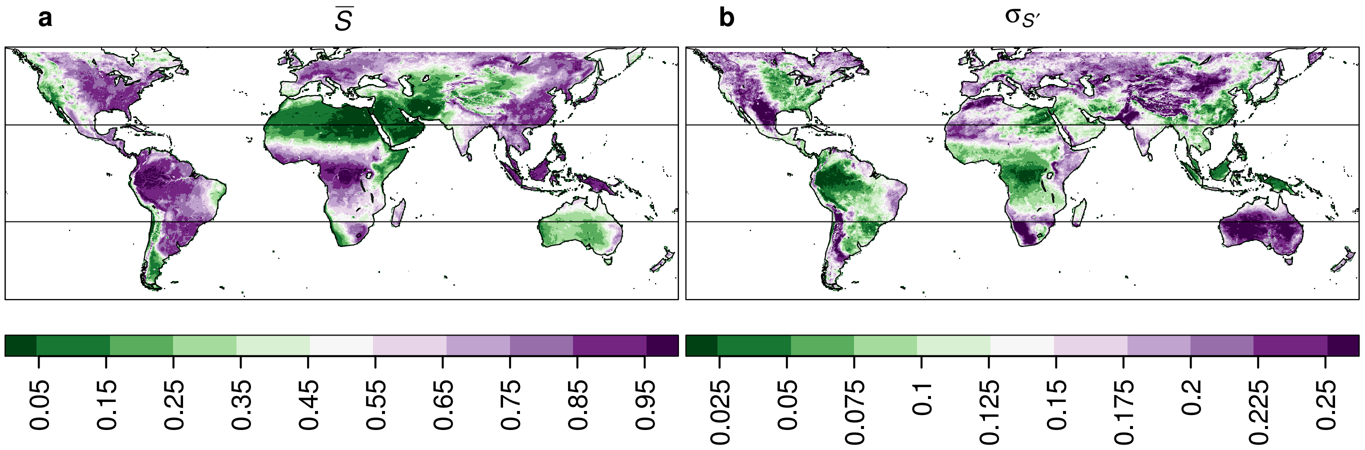

Supplementary Figure 9 | Properties of S from GLEAM. (**a**) Mean S and (**b**) standard deviation of S' from $\mathrm{GLEAM_C}$. $\mathrm{GLEAM_T}$ and $\mathrm{GLEAM_P}$ display similar patterns.

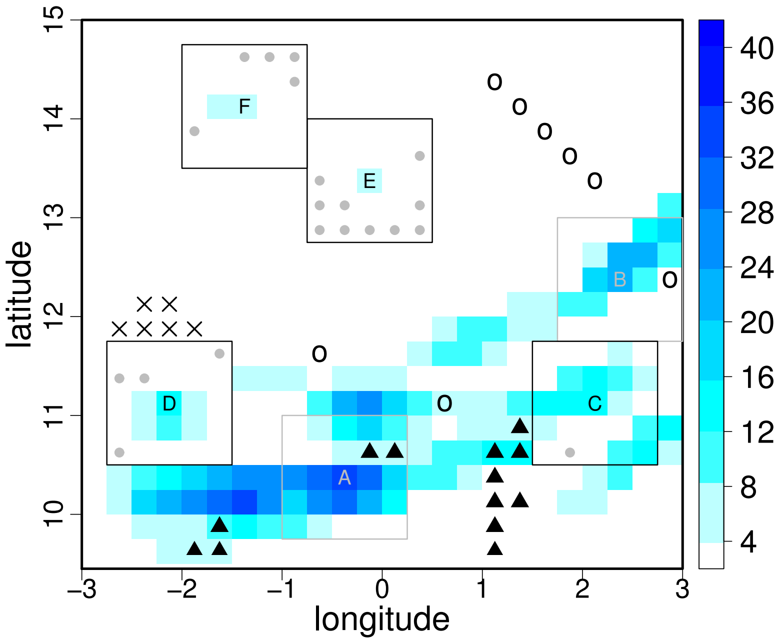

Supplementary Figure 10 | Example events in West Africa, on June 28, 2006. Background color indicates total afternoon precipitation in mm (12:00−24:00), black symbols indicate grid cells excluded because of (crosses) morning precipitation (> 1 mm), (triangle) topography gradients and (circles) water bodies. Events included in (excluded from) the computations are denoted by black (grey) squares. The center of each event (Lmax) is denoted with a letter; grey dots indicate Lmin. When two or more event boxes overlap, only the event with largest precipitation at Lmax is retained. Thus, a number of maxima are not interpreted as events. A total of six events are detected, four of which are included in the computation. Events "A" and "B" are not included as they include topography features or water body, respectively.

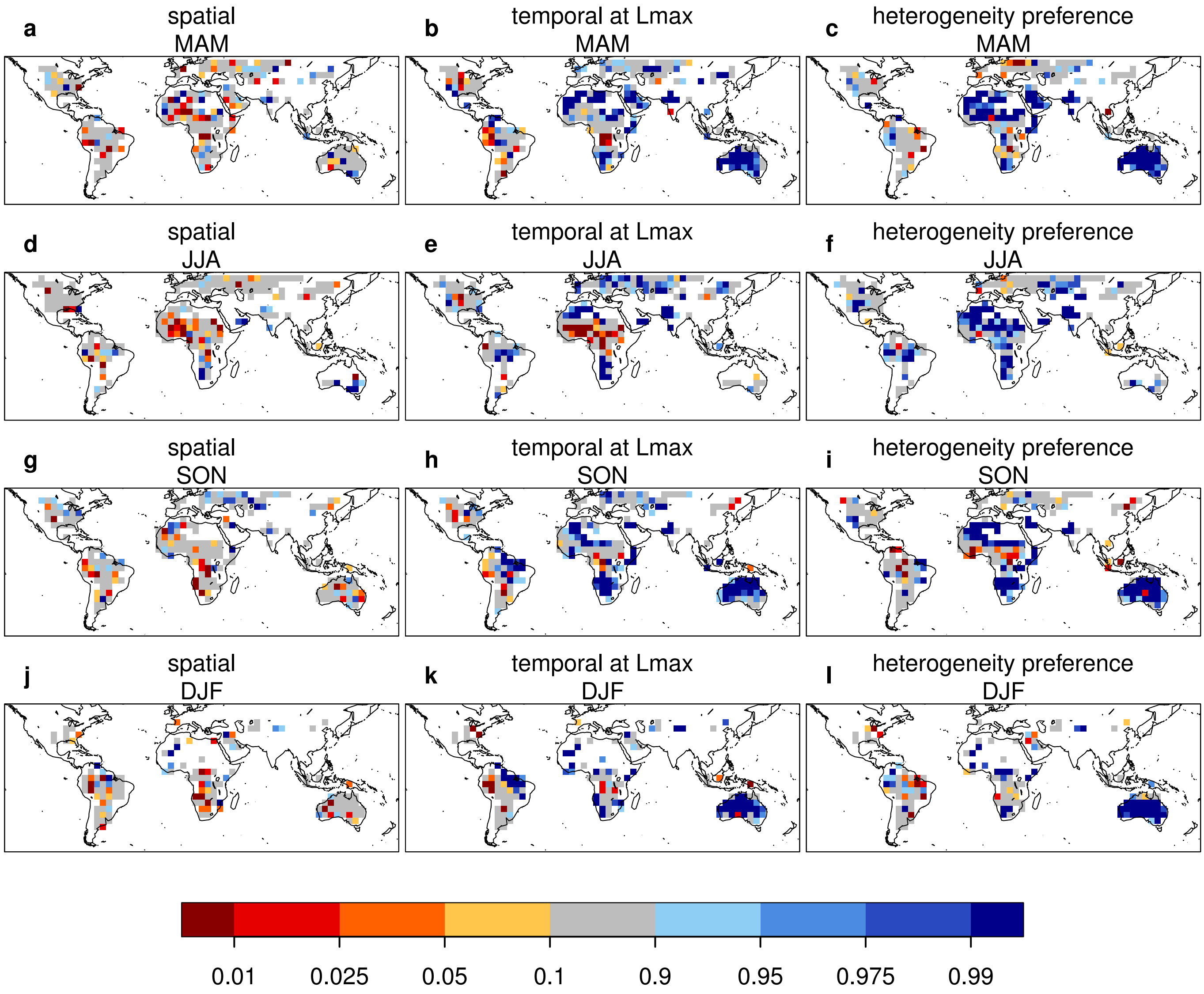

Supplementary Figure 11 | Seasonality in the coupling metrics for CMORPH precipitation and total soil moisture stress (S') from GLEAM$_C$. (**a,d,g,j**) spatial metric $\delta_e(Y^s)$ (as in Fig. 1a), (**b,e,h,k**) temporal metric at Lmax $\delta_e(Y^t)$ (as in Fig. 1b), (**c,f,i,l**) heterogeneity metric $\delta_e(Y^h)$ (as in Fig. 1c). A reduced threshold of 15 events is adopted.

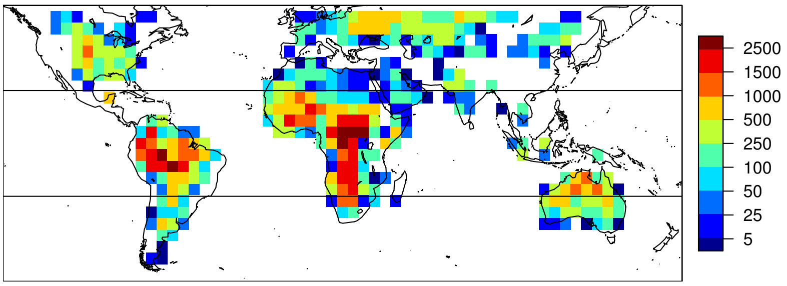

Supplementary Figure 12 | For each 5° box, number of events included in the analysis (i.e., after removal of events with morning precipitation, topography or water bodies) for the CMORPH−GLEAM$_C$ combination. Horizontal black lines indicate the latitudes at which different months are included in the analysis (see Methods). White boxes indicate no event, mostly related to strong topography features. A similar number of events is detected in other datasets.

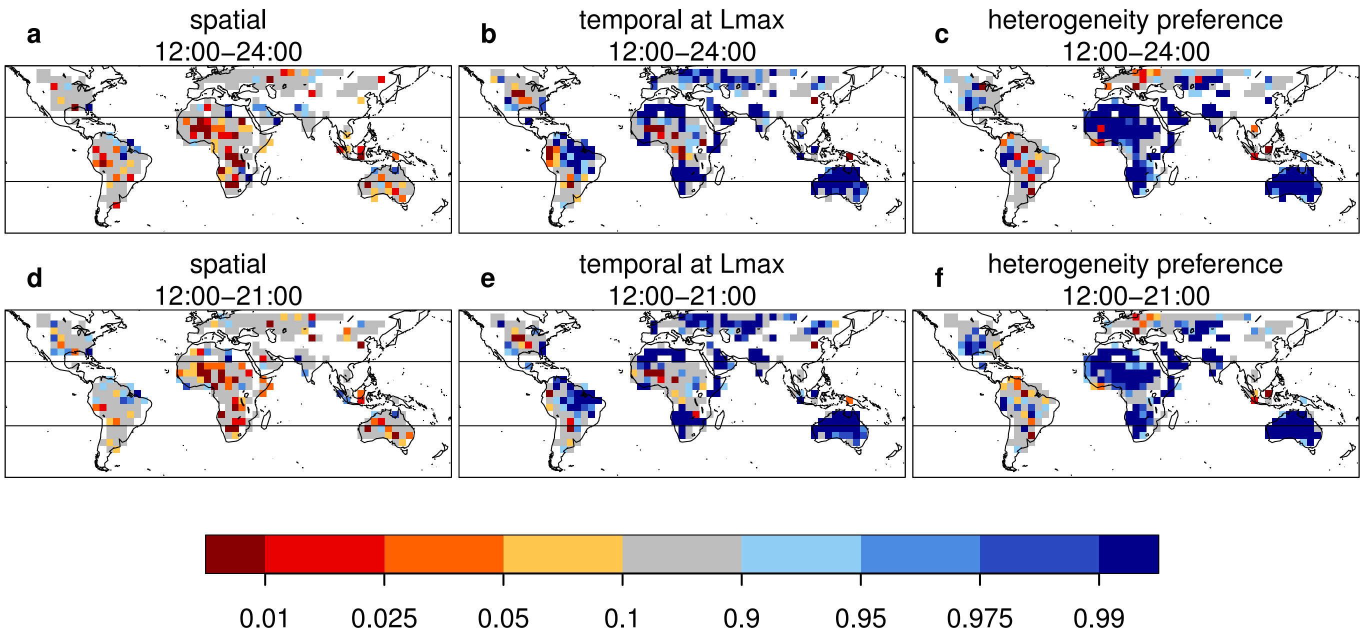

Supplementary Figure 13 | Sensitivity of the metrics to the time definition of afternoon rainfall, for CMORPH−GLEAM$_\mathbf{C}$. (**a,b,c**) 12:00−24:00, (**d,e,f**) 12:00−21:00. (**a,d**) Spatial metric from Fig. 1a, (**b,e**) temporal metric from Fig. 1b, (**c,f**) heterogeneity sensitivity metric from Fig. 1c.

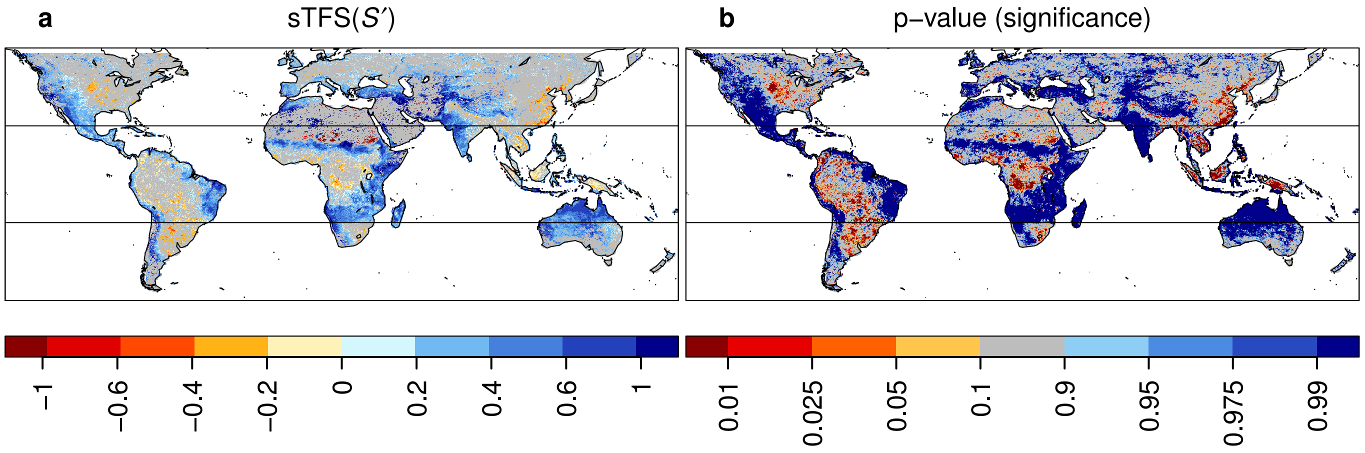

Supplementary Figure 14 | Scaled, simplified triggering feedback strength (sTFS) for CMORPH−GLEAM$_\mathbf{C}$. (**a**) sTFS(S'), (**b**) p-value (significance). The definition of sTFS is provided in the Supplementary Discussion. Horizontal black lines indicate the latitudes at which different months are included in the analysis (see Methods).

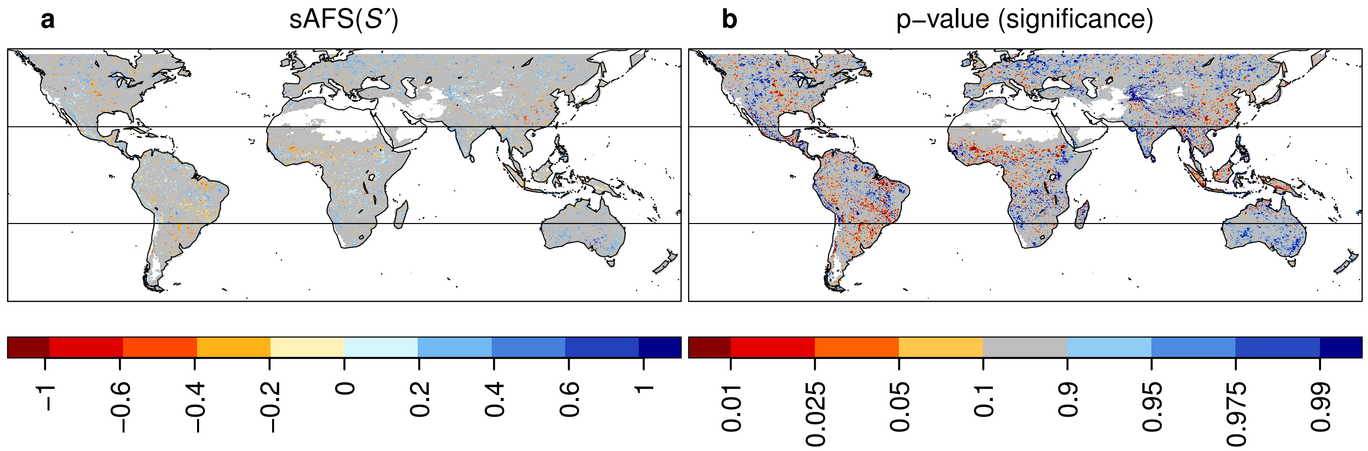

Supplementary Figure 15 | Scaled, simplified amplification feedback strength (sAFS) for CMORPH−GLEAM$_{\mathbf{C}}$. (**a**) sAFS(S'), (**b**) p-value (significance). The definition of sAFS is provided in the Supplementary Discussion. Horizontal black lines indicate the latitudes at which different months are included in the analysis (see Methods).

**Variables**	**Dataset**	**Temporal resolution**	**Spatial resolution**	**Reference**
Surface soil moisture	NASA-LPRM (AMSR-E)	night-time overpass	0.25°	Ref. 2
Vegetation optical depth	NASA-LPRM (AMSR-E)	daily	0.25°	Ref. 2
Precipitation	CMORPH v1.0	3 h	0.25°	Methods and ref. 3
Precipitation	PERSIANN	3 h	0.25°	Methods and ref. 4
Precipitation	TRMM 3B42 v7	3 h	0.25°	Methods and ref. 5
Net radiation	CERES_SYN1deg Ed3A	3 h	1°	Ref. 6
Air temperature	ERA-Interim	3 h (incl. forecast)	0.75°	Ref. 7

Supplementary Table 1 | Forcing datasets for GLEAM. Daily aggregates are computed locally to match the before-noon estimate (i.e., from 09:00 on previous day to 09:00 at present day to get 09:00 S and S_s).

**Variables**	**Dataset**	**Temporal resolution**	**Spatial resolution**	**Reference**
Precipitation	GPCP_1DD v1.2	daily	1°	Ref. 8
Precipitation (when GPCP missing)	ERA-Interim	3 h (incl. forecast)	0.75°	Ref. 7
Net radiation	ERA-Interim	3 h (incl. forecast)	0.75°	Ref. 7

Supplementary Table 2 | Datasets used to fill GLEAM input from Supplementary Table 1. Note that temperature data from ERA-Interim does not contain any gap.

Supplementary Discussion

Results with alternative datasets

A total of 12 dataset combinations are used for the analysis, combining three precipitation datasets (CMORPH, TRMM, PERSIANN) and four soil moisture datasets (GLEAM driven by our three precipitation datasets, and AMSR-E). For GLEAM soil moisture estimates, surface soil moisture stress over the bare soil fraction (S_s) is also included (in addition to total soil moisture stress S), since these may more directly relate to satellite soil moisture from AMSR-E. Results for all 12 dataset combinations are presented for our three metrics ($\delta_e(Y^s)$, $\delta_e(Y^t)$, $\delta_e(Y^h)$).

Spatial metric Supplementary Fig. 1 displays the results for the spatial metric from ref. 1, $\delta_e(Y^s)$ where $Y^s = S'_{\mathrm{Lmax}} - S'_{\mathrm{Lmin}}$, as well as our results with S from GLEAM (see also Fig. 1a) and with Θ_{top} from AMSR-E, while Supplementary Fig. 2 displays the same results for S_s from GLEAM.

The patterns are sensitive to both the soil moisture and precipitation datasets. The signal is strongest with TRMM and weakest with PERSIANN, consistently with ref. 1. The stronger signal with TRMM is due to larger soil moisture differences between event and non-event days. $\mathrm{GLEAM_T}$ also leads to a stronger signal than the other two GLEAM estimates. AMSR-E leads to a weak signal, likely because of data quality issues, while the assimilation procedure in GLEAM filters out unrealistic AMSR-E data (see also ref. 1, who apply strict data quality filter). For GLEAM, results with S_s (Supplementary Fig. 2) are less statistically significant than with S (Supplementary Fig. 1), emphasizing that S_s is not always representative of the actual soil moisture stress. This might also explain part of the differences with ref. 1 and highlights the advantage of considering the whole root zone.

Temporal metric The multi-dataset temporal results at Lmax ($\delta_e(Y^t)$) are displayed in Supplementary Fig. 3 for S from GLEAM (see also Fig. 1b) and Θ_{top} from AMSR-E, and in Supplementary Fig. 4 for S_s from GLEAM. Similarly to the spatial metrics results, the patterns exhibit some variability as a response to the choice of dataset, but the dominance of positive temporal relationships remains in all combinations. The various combinations also display some agreement in the few regions with negative relationships.

Heterogeneity metric The multi-dataset results for the sensitivity of rainfall to soil moisture heterogeneity ($\delta_e(Y^h)$) are displayed in Supplementary Fig. 7 for S from GLEAM (see also Fig. 1c) and Θ_{top} from AMSR-E, and in Supplementary Fig. 8 for S_s from GLEAM.

All dataset combinations show a clear dominance of more heterogeneous conditions than expected for precipitation events, although with slightly different patterns from different dataset combinations. This suggests that precipitation events might generate following events via the spatial feedback mechanism, and thereby leading, on a large scale, to a positive feedback, as previously proposed by ref. 9.

Temporal analysis from different locations

For temporal results, we have used soil moisture at Lmax (Fig. 1b). However, the overall soil moisture conditions in a larger area might be more representative of processes such as moisture recycling. Therefore, we here repeat the temporal analysis but using soil moisture averaged over Levt, the 5 × 5 grid cells (i.e., 1.25°) surrounding Lmax (see Methods), instead of soil moisture at Lmax. Supplementary Figure 5 displays the corresponding results for the 12 dataset combinations and can be compared to Supplementary Fig. 3. Results are roughly similar, indicating that the overall soil moisture conditions, rather than the condition at Lmax alone, might relate to afternoon precipitation. This supports the hypothesis of moisture recycling, although the various effects are difficult to explicitly disentangle, in particular the role of precipitation persistence. Note that moisture recycling is expected to act on time scales of days, not investigated here. Nonetheless, analyses using S' from the previous day (not shown) leads to similar (though weaker) results, supporting this mechanism.

Similarly, Supplementary Fig. 6 displays results using S' at Lmin, the location of rainfall minimum within Levt. Soil moisture at this location is clearly wetter for event cases than for non-event cases. This can be expected, since it is the case for S'_{Lmax} and since the spatial metric shows that S'_{Lmax} tends to be smaller than S'_{Lmin} on event days, but it again highlights that the conditions before precipitation events are often wet.

Seasonality in spatial and temporal metrics

The seasonality in the metrics is presented in Supplementary Fig. 11 for CMORPH-$\mathrm{GLEAM_C}$, and highlights some season-dependent patterns. In particular, more negative temporal relationships are found in some regions for some seasons, typically rainy seasons with a larger number of events available compared to the seasons merged in the remaining of our analysis.

Properties of soil moisture data

Supplementary Figure 9 displays the mean and standard deviation of the evaporative stress (S) from GLEAM. Regions with large variability correspond to transitional regions between wet and dry climates (high and low mean S, respectively), where soil moisture is limiting but there is enough moisture supply to allow substantial variability.

Alternative temporal metric: the simplified triggering feedback strength

Our temporal metric based on precipitation event detection differs from traditional, time-series based analyses. Here, we display such a metric, the triggering feedback strength from ref. 10, which quantifies the relationship between before-noon EF and afternoon precipitation occurrence as $\mathrm{TFS} = \sigma_{\mathrm{EF}} \frac{\partial \Gamma(r)}{\partial \mathrm{EF}}$, where $\Gamma(r)$ is the probability of afternoon rainfall ($r > 1$ mm) and EF is before-noon (09:00−12:00) evaporative fraction ($\mathrm{EF} = \lambda E/(H + \lambda E)$ where H and $\lambda \mathrm{E}$ are surface sensible and latent heat fluxes, respectively). We replace EF by S and we use anomalies from the seasonal cycle (S'). In addition, we scale TFS by the mean afternoon precipitation occurrence ($\overline{\Gamma(r)}$) to allow for comparison between regions with different precipitation regimes. Thus, we define a simplified triggering feedback strength,

$$\mathrm{sTFS}(S') = \frac{\sigma_{S'}}{\overline{\Gamma(r)}} \frac{\partial \Gamma(r)}{\partial S'} , \tag{1}$$

where $\sigma_{S'}$ is the standard deviation of S' (using 09:00−12:00 values from each day), and $\Gamma(r)$ is the probability of afternoon rainfall ($r > 1$ mm for the 12:00−24:00 time period). The numeric computation uses two bins, and significance is tested by means of 1000 bootstraps samples as in ref. 11. In addition, the original computation from ref. 10, binned on variables relating to atmospheric humidity and stability, is replaced by the simpler computation from Eq. 1. Days with morning precipitation exceeding 1 mm in any neighbouring grid cells in a box of 1.25° surrounding each grid cell are excluded from the computation. Supplementary Fig. 14 displays sTFS(S') and its p-values relative to the null distribution obtained from bootstrapping. Note that no topography- or water-related filter is applied. Comparing these results with our temporal metric (e.g., Fig. 1b from the main text), it is interesting to note that the significance (p-values) depicts similar regions of positive and negative temporal relationships.

While our methodology focuses on the impact of soil moisture on precipitation occurrence, ref. 10 also introduced a metric quantifying the relationship between before-noon EF and afternoon precipitation amounts: The amplification feedback strength (AFS), restricted to days with afternoon rain (> 1 mm). Supplementary Figure 15 displays sAFS, a simplified AFS formulation similar to what sTFS is to TFS. No strong relationship is found anywhere, suggesting that the local impact of soil moisture on rainfall amounts is negligible and consistently with the findings from ref. 10 over North America. Nonetheless, we note that the accuracy of rainfall amounts has been shown to be low relative to the accuracy of rainfall occurrence [12], which may prevent the exclusion of such local impacts on precipitation amounts.

Supplementary References

[1] Taylor, C. M., de Jeu, R. A. M., Guichard, F., Harris, P. P. & Dorigo, W. A. Afternoon rain more likely over drier soils. *Nature* **489**, 423–426 (2012).

[2] Owe, M., de Jeu, R. & Holmes, T. Multisensor historical climatology of satellite-derived global land surface moisture. *J. Geophys. Res.* **113**, F01002 (2008).

[3] Joyce, R. J., Janowiak, J. E., Arkin, P. A. & Xie, P. CMORPH: A Method that Produces Global Precipitation Estimates from Passive Microwave and Infrared Data at High Spatial and Temporal Resolution. *J. Hydrometeorol.* **5**, 487–503 (2004).

[4] Hsu, K.-l., Gao, X., Sorooshian, S. & Gupta, H. V. Precipitation Estimation from Remotely Sensed Information Using Artificial Neural Networks. *J. Appl. Meteorol.* **36**, 1176–1190 (1997).

[5] Huffman, G. J. *et al.* The TRMM Multisatellite Precipitation Analysis (TMPA): Quasi-Global, Multiyear, Combined-Sensor Precipitation Estimates at Fine Scales. *J. Hydrometeorol.* **8**, 38–55 (2007).

[6] Wielicki, B. A. *et al.* Clouds and the Earth's Radiant Energy System (CERES): An Earth Observing System Experiment. *Bull. Am. Meteorol. Soc.* **77**, 853–868 (1996).

[7] Dee, D. P. *et al.* The ERA-Interim reanalysis: configuration and performance of the data assimilation system. *Quart. J. Roy. Meteor. Soc.* **137**, 553–597 (2011).

[8] Huffman, G. J. *et al.* Global Precipitation at One-Degree Daily Resolution from Multisatellite Observations. *J. Hydrometeorol.* **2**, 36–50 (2001).

[9] Taylor, C. M. *et al.* Frequency of Sahelian storm initiation enhanced over mesoscale soil-moisture patterns. *Nature Geosci.* **4**, 1–4 (2011).

[10] Findell, K. L., Gentine, P., Lintner, B. R. & Kerr, C. Probability of afternoon precipitation in eastern United States and Mexico enhanced by high evaporation. *Nature Geosci.* **4**, 434–439 (2011).

[11] Guillod, B. P. *et al.* Land-surface controls on afternoon precipitation diagnosed from observational data: uncertainties and confounding factors. *Atmos. Chem. Phys.* **14**, 8343–8367 (2014).

[12] Tian, Y. *et al.* Component analysis of errors in satellite-based precipitation estimates. *J. Geophys. Res.* **114**, D24101 (2009).
